# Streamlining Quantification and Data Harmonization of Polychlorinated Alkanes Using a Platform-Independent Workflow

**DOI:** 10.1021/acs.est.5c04928

**Published:** 2025-10-09

**Authors:** Idoia Beloki Ezker, Bo Yuan, Anders Røsrud Borgen, Jiyan Liu, Yawei Wang, Thanh Wang

**Affiliations:** † Department of Physics, Chemistry and Biology (IFM), 4566 Linköping University, 581 83 Linköping, Sweden; ‡ Department of Chemistry, 8018 Norwegian University of Science and Technology, 7491 Trondheim, Norway; § Environmental Chemistry and Health Effects, NILU, 2007 Kjeller, Norway; ∥ State Key Laboratory of Environmental Chemistry and Ecotoxicology, Research Center for Eco-Environmental Sciences, 12381 Chinese Academy of Sciences, Beijing 100085, China; ⊥ School of Environment, Hangzhou Institute for Advanced Study, University of Chinese Academy of Sciences, Hangzhou, Zhejiang 310000, China; # Department of Thematic Studies − Environmental Change, Linköping University, 581 83 Linköping, Sweden

**Keywords:** polychlorinated alkanes, chlorinated paraffins, quantification, data harmonization, persistent organic pollutants, analytical method

## Abstract

Reliable quantification of polychlorinated alkanes (PCAs) remains a major challenge, hindering environmental research across diverse matrices. Each sample can contain over 500 homologue groups, collectively producing >1000 *m*/*z* ratios that require interference checks. High-resolution mass spectrometry methods vary in ionization signals and data formats and require specialized algorithms for quantification. CPxplorer streamlines data processing through the integration of three modules: (1) CPions generates target ion sets and isotopic thresholds for compound identification into the next module; (2) Skyline performs instrument-independent data integration, interference evaluation, and homologue profiling; and (3) CPquant deconvolves homologues and reports concentrations using reference standards and homologue profiles from Skyline. Evaluation of the workflow with NIST-SRM-2585 dust and ERM-CE100 fish tissue material yielded comparable results across raw data formats from different instruments. Further applications of CPxplorer across diverse matrices, including indoor dust, organic films, silicone wrist bands, and food samples, demonstrated the usefulness in biological and environmental monitoring. Compared to existing tools limited to qualitative detection, CPxplorer enables quantitative outputs, reduces processing time, and expands functionality to PCA-like substances (e.g., BCAs) and PCA degradation products (e.g., OH-PCAs). CPxplorer reduces learning barriers, empowers users to quantify PCAs across various analytical instruments, and contributes to generating comparable results in the field.

## Graphical abstract

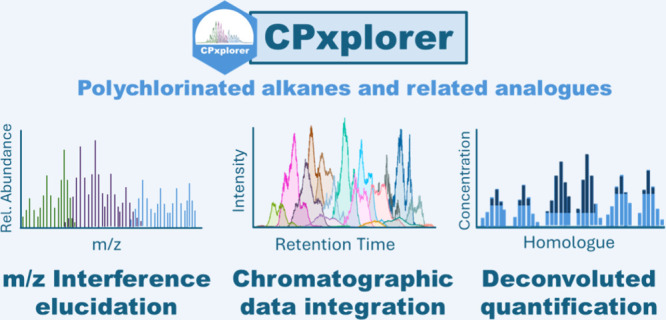

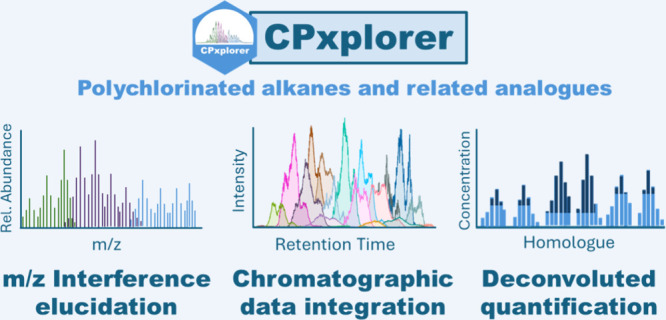

## 1 Introduction

Polychlorinated alkanes (PCAs, C_*n*_H_2*n*+2–*x*_Cl_*x*_) are the main component of chlorinated paraffins (CPs) technical mixtures.^1^ CPs have a wide range of applications as flame retardants and plasticizers,^2^ and their cumulative production has exceeded 33 million metric tons since the 1930s.^3^ Consequently, PCAs are ubiquitous in the outdoor and indoor environments, and humans are constantly exposed to this complex contaminant group.^2^ The short carbon chain homologue groups (C_10_–C_13_), also named short-chain chlorinated paraffins (SCCPs), have persistent, bioaccumulative, and toxic (PBT) properties, hence their production and use were banned by the Stockholm Convention on POPs (persistent organic pollutants) in 2017.^4^ Recently, MCCPs (medium- chain CPs, C_14_–C_17_) have also faced a ban,^5^ and LCCPs (long-chain CPs, C_18_–C_30_) are also suspected to be PBT but remain unregulated.^3^ This lack of regulation on CPs can be attributed to the limited toxicological and fate data, stemming from the complex nature of PCA analysis.^6^

The complexity of PCA analysis is heightened by the large number of isomers, homologue groups (expressed commonly as PCAs-C_*x*_Cl_*y*_),^1^ and chain length congener groups (PCAs-C_*x*_)^1^ that chromatographic techniques lack the resolution to fully resolve.^6^ Consequently, the chromatographic signals result in broad “humps”^7^ that conventional data analysis software struggle to accurately identify. Additionally, the *m*/*z* values of these homologue groups significantly overlap, with the *m*/*z* ranges extending from 200 to 750, 250–800, and 450–950 for 34 PCAs-C_10–13_Cl_3–13_, 47 PCAs-C_14–17_Cl_3–15_, and 156 PCAs-C_18–30_Cl_3–15_ homologue groups, respectively. The isotopic patterns overlap and interfere with each other, complicating the PCA analysis when using low-resolution mass spectrometry (LRMS).^7,8^ This highlights the necessity for high-resolution mass spectrometry (HRMS) with a minimum of 20 000 resolving power to achieve more accurate results. However, the choice of HRMS instruments also impacts PCA homologue identification, as different chromatographic and ionization techniques tend to favor specific chlorination levels or carbon chain lengths.^9,10^

Instrumental limitations in PCA analysis hamper the isomeric differentiation and complicate the quantification process as the accurate matching of standards and samples is impeded.^11^ In fact, existing standards do not reflect the true PCA distribution,^12,13^ requiring mathematical tools for semiquantification.^11,14,15^ This leads to varying data treatment methods across laboratories, complicating interlaboratory comparisons.^16,17^ The most commonly applied method was described by Bogdal et al.^11^ and relies on a mathematical algorithm to deconvolute PCA patterns in a sample from combinations of different standards. However, setting up this method is tedious and challenging to comprehend, making it difficult for most laboratories to implement as no software currently automates the process.

Currently, there are few open-access tools that offer data analysis of PCAs from mass spectrometric measurements, which complicates data collection for proper risk assessments, both in environmental contexts and in areas including food and feed safety. Available tools such as CP-Seeker offer a solution for automatizing the chromatographic peak integration,^18^ while CP-Hunter aids in exploring mass spectral interferences.^19^ However, no tool has yet integrated the quantification step into the workflow. Furthermore, the use of different tools and methods across laboratories can results in nonharmonized values,^17^ hindering the establishment of exposure reference values for PCAs. Moreover, assessing multiple human exposure pathways and the diversity of sample matrices presents additional challenges. The lack of suitable workflows to address these complexities further impedes the ability to accurately evaluate risks and exposure levels across various environmental contexts.^12^

To address this, we have developed CPxplorer, an open-source solution that rapidly processes raw data and reliably quantifies a large number of samples, featuring detailed manuals and tutorials, and intuitive graphical user interfaces (GUIs) to support users of all experience levels. CPxplorer tackles mass spectral overlaps of PCAs and other byproducts in CP mixtures, offering a rapid data integration solution and comprehensive guidance on peak selection. It also provides quantified PCA levels by automating the script of Perkons et al.,^20^ which applies the modified deconvolution method described by Bogdal et al.^11^ The method was evaluated by analyzing the NIST indoor dust reference sample (SRM 2585, National Institute of Standards and Technology) using various chromatographic and mass spectrometric techniques along with different standards, including standard mixtures, single-chain standards, and technical mixtures. To further confirm the method, CPxplorer was used to quantify PCAs-C_10–13_ and PCAs-C_14–17_ from the certified fish tissue material, ERM-CE100. CPxplorer was then applied to quantify PCA levels in matrices commonly used to assess human exposure, such as indoor dust, indoor organic film (IOF), silicone wristbands (SWBs), and food. Additionally, CPxplorer was used to investigate the presence of CP mixture byproducts, such as polychlorinated olefins (PCOs) and bromochloro alkanes (BCAs), as well as their transformation products. Overall, CPxplorer introduces a novel framework for automatizing PCA quantification and streamlining data harmonization with potential implications for enhanced risk assessment workflows.

## 2 Experimental Procedures

### 2.1 CPxplorer Overview

CPxplorer presents a complete solution for PCA analysis and quantification. The workflow that we developed contains three integrated modules, CPions, Skyline,^21^ and CPquant, which facilitate the quantification of large data sets in a short period of time. Depicted in Figure 1A, CPions is a tool for the exploration of the CP mixture mass spectral interferences and can generate a list of selected targeted ions, such as those that have noninterfering *m*/*z* values. This target ion list is then imported into Skyline (Figure 1B), which allows for the synchronized peak integration of large sample batches without the need for prior data conversion. Finally, the exported report from Skyline is directly uploaded into CPquant (Figure 1C), which provides the quantification results of PCAs by deconvoluting the contribution of the different homologue groups.^11,20^

**Figure 1:**
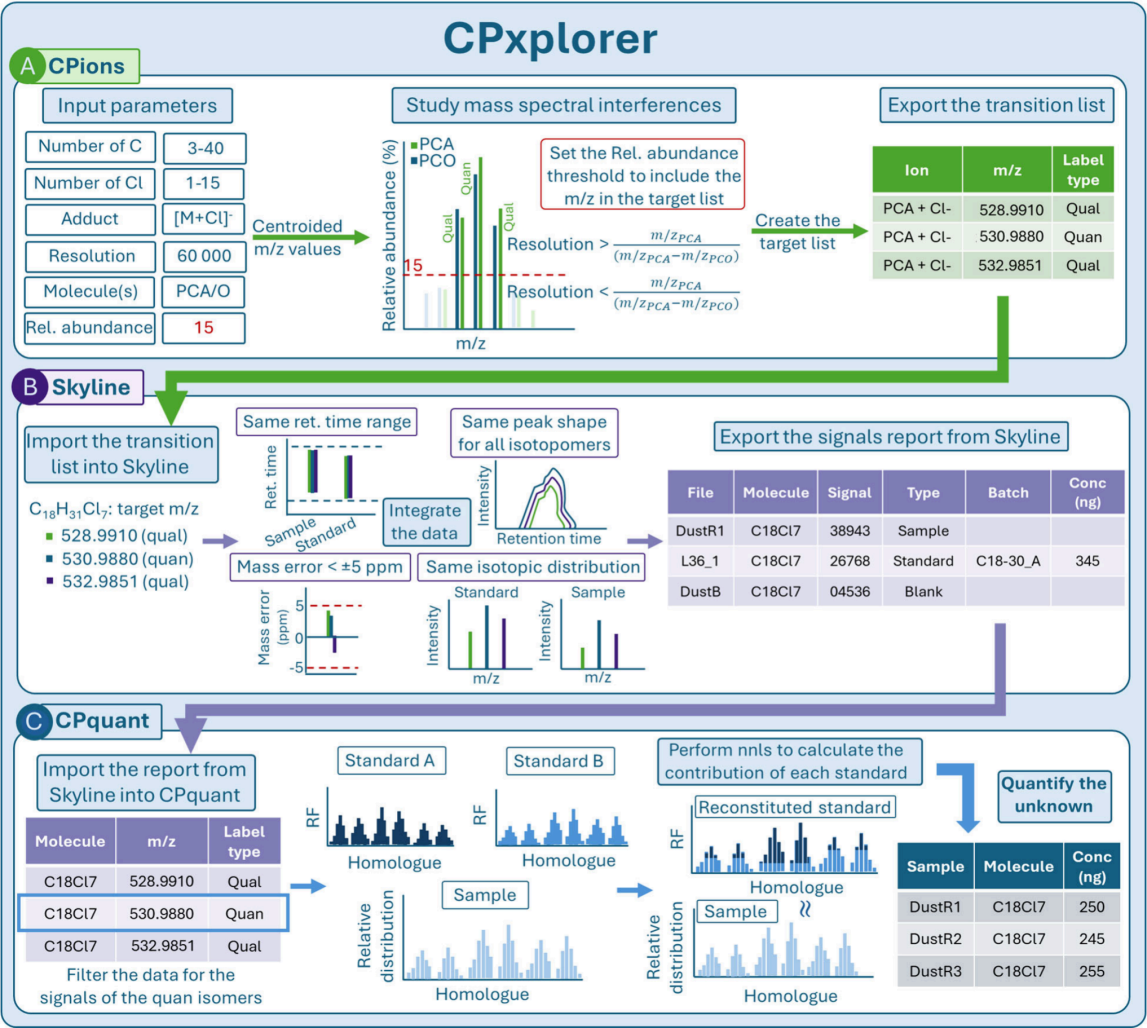
Scheme of the CPxplorer workflow. From top to bottom, (A) CPions is an app for the generation of target ions list and investigates potential mass interferences. (B) Skyline is an openly available software for raw mass spectrometric data integration^21^ and operates on the target list created by CPions. (C) CPquant is an app that performs quantification of PCAs in the samples and blanks based on the integration results from Skyline. Detailed descriptions of each module can be found in Sections S8–S10. Note: “Molecule” the is the term used by Skyline, which refers to homologue group.

### 2.2 Workflow Evaluation

The method was applied to the analysis of PCAs-C_10–30_ in the 2585 SRM from NIST. The SRM-2585 has been broadly used for the study of organic contaminants in house dust, including PCAs, whose measured values at average concentrations were 7.58 (±0.43) and 16.4 (±2.1) μg/g for ∑PCAs-C_10–13_ and ∑PCAs-C_14–17_, respectively.^22^ In order to provide a comprehensive evaluation of CPxplorer, different instrumental techniques and ionization modes were applied, including gas chromatography (GC)–negative chemical ionization (NCI)–quadrupole time-of-flight (qToF), GC-NCI-Orbitrap, liquid chromatography (LC)–electrospray ionization (ESI)–qToF, LC-ESI-Orbitrap, LC–atmospheric pressure chemical ionization (APCI)–Orbitrap, and direct injection (DI)–APCI–Orbitrap. Additionally, the fish tissue ERM-CE100 was analyzed by GC-NCI-qToF and CPxplorer was applied for PCA data treatment and quantification. ERM-CE100 is a recently available certified material for PCAs-C_10–13_ (31 ± 9 and 23 ± 7 μg/kg, depending on the calibrant used) and PCAs-C_14–17_ (44 ± 17 μg/kg).^23^ Detailed information on NIST-SRM-2585 and ERM-CE100 can be found in Sections S1 and S2 and Table S1.

For the quantification of PCAs, different standard mixtures and several combinations were used to ensure an exhaustive evaluation of CPxplorer. The following single-chain standard mixtures were purchased from Chiron AS: C_10_ (52.5% and 58.4% Cl), C_11_ (52.3% and 57.7% Cl), C_12_ (53.8% and 57.3% Cl), C_13_ (45.9% and 60% Cl), and C_14_ (49.2% and 58.7% Cl), C_15_ (47.7% and 59.3% Cl), C_16_ (51.5% and 58.4% Cl), and C_17_ (56.3% Cl). PCA standard mixtures from Dr. Ehrenstorfer (Augsburg, Germany) containing PCAs-C_10–13_ with 51.5%, 55.5%, and 63.0% chlorine content, PCAs-C_14–17_ with 42%, 52%, and 57% chlorine content, and PCAs-C_18–30_ with 36% and 49% chlorine content were purchased. Complementarily, the technical mixtures Uniclor40 from Neville Chemical Co (USA), Paroil CW 40 from Dover Chemical Corporation (USA), Cloparin 49 from Caffaro (Italy), Hüls 70C and 40N from Hüls AG (Germany), Witaclor 149 and 549 from Dynamit Nobel AG (Germany), and Cereclor S52 from INEOS Chlor, Ltd. (UK) were used. The calibration curves were built at three to five concentration points of each standard mixture.

### 2.3 Environmental Samples

The applicability of CPxplorer was tested as a tool for the rapid assessment of human exposure to PCAs. Indoor dust and IOF samples were collected in parallel in a Swedish household in May 2023. Complementarily, a participant living in this household wore an SWB for over a week. Pooled vegetables and meat samples from a Swedish market basket study were also analyzed. These represented some of the most consumed items from the Swedish food market, with a consumption of 245 and 194 g/person/day per capita of vegetables and meat, respectively.^24^ All samples were analyzed by LC-ESI-qToF (Agilent 6546); detailed information on sample preparation can be found in Sections S3–S6.

In order to further understand the implications of human exposure to CPs and further extend the applications of CPxplorer, PCOs, Phase I degradation products of PCAs, and BCAs were also investigated. For PCA degradation products, rice plant roots (*Oryza sativa* Japonica cv. Nipponbare) previously exposed to 1,2,5,6,9,10-C_10_H_16_Cl_6_ were analyzed. The data were originally acquired by Chen et al.^25^ using LC-ESI-Orbitrap (Section S7). PCOs and BCAs were investigated in previously described NIST-SRM-2585 indoor dust.

### 2.4 Data Treatment Procedure

The first step of CPxplorer involves the creation of a target analyte list which compiles the nonconflictive *m*/*z* values. This is performed by the module CPions, an in-house developed graphical user interface app based on the Shiny framework in R. The source code is available at the GitHub repository (https://github.com/WBS-TW/CPxplorer). Briefly, in CPions the centroided *m*/*z* values corresponding to the ions are generated based on the EnviPat package.^26^ As depicted in Figure 1, the *m*/*z* values that are not expected to overlap at the applied mass resolving power are considered for the analyte target list. The app is flexible and enables the study of a large variety of adducts of PCAs, BCAs, PCOs, and degradation products. A visualization of the GUI of CPions can be seen in Figure S2, and a detailed description of CPions can be found in Section S8.

The *m*/*z* value list generated in CPions can then be imported to Skyline to generate the extracted ion chromatograms (EIC) from the raw data (Figure 1). Skyline is an open-source software for quantitative analysis of mass spectrometric data, which supports .d, .wiff, .qgd, .raw, and .mzml file extensions from the major vendors Agilent and Bruker, Sciex, Shimadzu, Thermo, and Waters.^27^ Peak detection is performed within a user-defined mass error window. The integration criteria include matching isotopologue peak shapes, similar isotope abundance patterns, and retention time ranges between the standard and the sample, confirming Gaussian distribution of profile MS data and ensuring mass error is within acceptable ranges (Figure 1). A visualization of the GUI of Skyline can be seen in Figure S3 together with a detailed description of the tool in Section S9.

Data integration reports from Skyline can be exported to a .csv or spreadsheet file, which can then be imported into CPquant for quantification. CPquant is another Shiny app in our CPxplorer package, which is mainly written by using the tidyverse grammar of the R programming language. The quantification values are obtained using the PCA homologue deconvolution method^11,20^ (Figure 1C). Specifically, the CPquant approach builds upon the R script from Perkons et al.,^20^ which performs the least-squares approximation by assuming two contributing standard mixtures and solving the best fit under a fixed total contribution. In contrast, our approach is based on the non-negative least-squares (nnls) R package and allows for the simultaneous use of all standards without imposing a fixed-sum constraint, enabling batch analysis rather than the single-sample quantification used in previous quantification schemes. This significantly enhances efficiency and scalability, making batch-scale quantification feasible; see details in Section S10. A visualization of the GUI of CPquant can be seen in Figure S4. Another advantage of CPquant is the output of QA/QC data to further ensure the reliability of the quantified results, such as the goodness of pattern fit (GoPF) of the deconvoluted patterns, signal normalization to an internal standard, blank subtraction, and graphical displays of the signal ratios between quantification and qualification ions.

## 3 Results and Discussion

CPxplorer greatly reduced data quantification time to process large data sets. CPquant offers automatization of the script from Perkons et al.,^20^ for mass spectral deconvolution method.^11^ This advancement represents a major step forward in PCA analysis, which, to date, has been intricate and highly time-consuming. Additionally, CPquant allows for blank signal subtraction, a critical need in PCA analysis due to their ubiquitous presence and notably elevated levels in procedural blanks.^28^ Likewise, the use of CPxplorer for chromatographic peak integration poses a substantial improvement in data harmonization. To date, signal selection has relied on researcher-defined criteria. CPxplorer, however, introduces a streamlined and consistent approach for peak selection, delivering results comparable to previously published studies (e.g., NIST SRM-2585, Figure 2). The module CPions provided a rapid and comprehensive understanding of the mass spectral interferences and generated a list of target compounds. This list ensured that the selected *m*/*z* values can be free from interference, providing additional confidence in peak selection and accurate identification of PCAs. The Skyline platform further enhanced this process with multivariable criteria, enabling simultaneous visualization of chromatograms, retention times, MS data, isotopic distributions, and mass errors. In addition, CPquant enhances data accuracy and consistency by automating and harmonizing quantification and QA/QC procedures. This comprehensive framework accelerated analysis while ensuring data quality and reliability in a few minutes.

**Figure 2:**
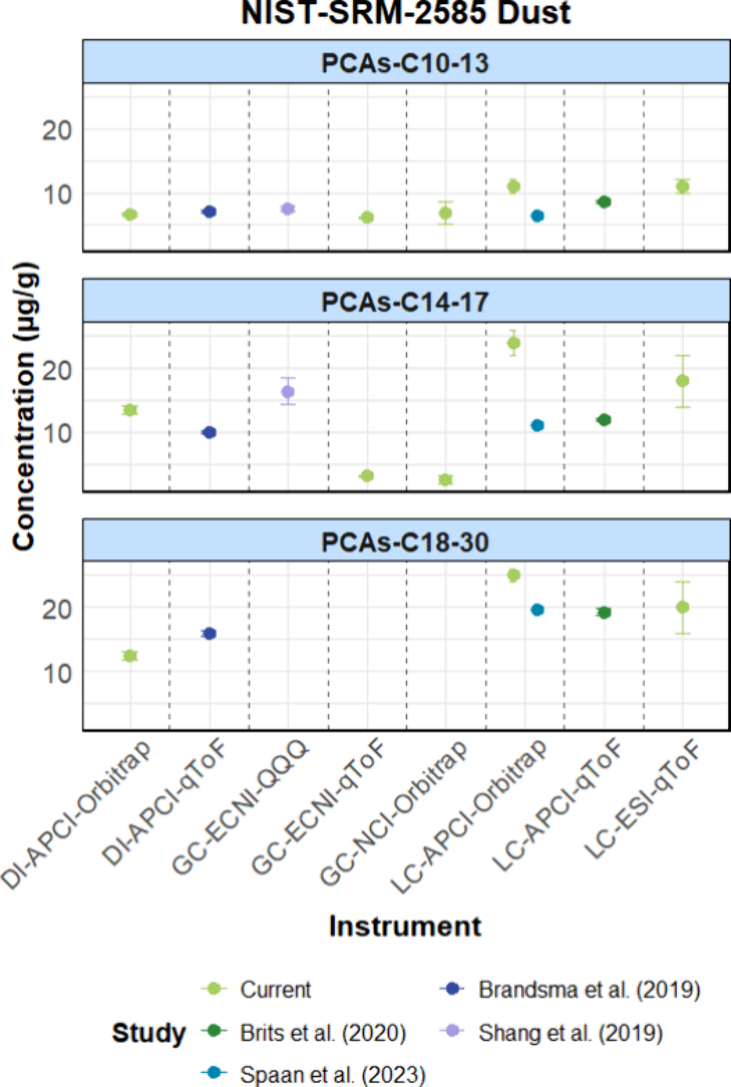
From top to bottom: ∑PCAs-C_10–13_, ∑PCAs-C_14–17_, and ∑PCAs-C_18–30_ levels (μg/g) in NIST-SRM-2585. The current study quantified the PCA levels by CPxplorer, displayed in light green of the online version. Note: [M + Cl]^−^ adducts of PCAs-Cl_4–15_ and [M – Cl]^−^ fragments of PCAs-Cl_6–11_ were detected when using LC-based techniques and GC-based techniques, respectively.

### 3.1 Evaluation of the CPxplorer Workflow

CPxplorer is presented as a standardized tool for PCA data quantification. To assess its suitability, data sets from a broad range of instruments were acquired and processed using the CPxplorer workflow. Given the diversity of instrumental capabilities across laboratories, a standardized tool must be capable of consistent performance across all data sets. However, full harmonization necessitates standardization at both the instrumental and data processing levels, with CPxplorer specifically addressing the latter. To support this, the influence of instrumentation on PCA responses was assessed, aiding in distinguishing the variability attributable to instrumentation from those introduced during data processing. Additionally, CPxplorer was further evaluated by comparing the performance of the current tool to established software, such as Trace Finder from Thermo Fisher and Mass Hunter from Agilent, as well as PCA-specific tools, including CP-Seeker,^18^ CP-Hunter,^19^ and the R-script published by Perkons et al.^20^

#### 3.1.1 Performance of CPxplorer with Diverse Instrumental Data

As depicted in Figure 2, CPxplorer provided quantified PCA levels for NIST-SRM-2585 comparable to those reported by previous studies.^22,29–31^ These results provided an open-access and more accurate solution for PCA analysis, significantly reducing the data processing time. The data were obtained by GC, LC, and DI coupled to ESI, APCI, and NCI-Orbitrap and qToF, showing also the usefulness of CPxplorer among different instruments and vendor file formats. This versatility of CPxplorer is particularly important in risk assessment, as the PCA pattern observed in the samples depends on the instrumental method used and may affect which homologues are identified as key drivers of toxicity.^9,10^ Furthermore, this flexibility is highly significant as it will enable the workflow to be implemented across a wide range of laboratories.

The GoPF (indicated by R^2^-values) of the PCA pattern in NIST-SRM-2585 calculated by CPquant and the measured signals ranged from 0.78 to 0.95, with 1.00 being a perfect match (Table S2). The quantified levels of ∑PCAs-C_10–13_ ranged from 6.1 ± 0.2 to 11 ± 1 μg/g; all results are compiled in Table S2. LC-based techniques, including LC-ESI-qToF and LC-APCI-Orbitrap, quantified slightly higher levels of ∑PCAs-C_10–13_, which may be attributed to the absence of blank subtraction in the data set. However, due to the lack of access to blank data from the different works, this possibility could not be further examined in the present study. The quantified levels of ∑PCAs-C_14–17_ resulted in 7-fold lower levels for GC-based (GC-NCI-Orbitrap, 2.5 ± 0.6 μg/g, and GC-ECNI-qToF, 3.1 ± 0.2 μg/g) compared to LC-based instruments (24 ± 2 and 18 ± 4 μg/g for LC-APCI-Orbitrap and LC-ESI-qToF, respectively), a difference that may also be explained by the blanks. This suggests that PCA-Cl_≤5_ homologues, which are not detected by GC-NCI techniques,^9^ may predominantly originate from blank contamination within the batch. Thus, the blanks may account for the higher levels of PCAs-C_14–17_ observed with LC-based techniques compared to GC-based techniques. See Figures S5A and S6A for the PCA-C_14–17_ patterns in NIST-SRM-2585 measured by LC and GC instrumentations.

CPions also illustrated the variability between instruments in ion interference analysis, where differences in targeted adducts further influence mass spectral overlap.^32^ When analyzing PCAs using GC-based techniques [PCA – Cl]^−^ fragments are commonly targeted, while with LC-based instrumentations [PCA + Cl]^−^ adducts are typically monitored.^9,10^ For instance, calculating at a resolution power of 20,000 and a relative abundance threshold above 50%, the [PCA – ^35^Cl]^−^ and [PCO – ^37^Cl]^−^ ions of the parent formulas C_12_H_19_Cl_7_ and C_12_H_17_Cl_7_, respectively, were found to overlap, while [PCA + ^35^Cl]^−^ and [PCO + ^37^Cl]^−^ were not. This mismatch in the target ion selection between techniques might have contributed to the disparity in the final quantified results due to the different ionization efficiencies. In addition, unresolved mass spectral signals can appear as a single peak in the chromatogram, as PCA peaks often span several minutes and lack distinct Gaussian shapes. This overlap can lead to over- or underestimation of concentration and reduce quantification accuracy.

PCA patterns in NIST-SRM-2585 dust were also influenced by instrumentation at the congener and homologue levels. The LC-based techniques showed the highest relative abundance for the PCA-C_14_ congeners, while the results obtained when using GC separation were dominated by the shorter chain congeners, PCAs-C_12_. However, adduct formation was not found to be the only factor driving the discrepancy between instruments. The higher volatile character of short-chain compared to the medium- or long-chain PCAs makes the GC more sensitive to PCAs-C_10–13_. The results acquired by the GC-NCI-Orbitrap described a PCA homologue pattern comparable to the one reported by Spaan et al.,^30^ where PCAs-C_12_ were the most abundant congeners. Contrarily, when using GC-NCI-qToF the PCA-C_10_ congeners were identified as the dominant chain length group. A closer examination of homologue patterns reveals that the results acquired by the qToF exhibited the highest relative abundance at a lower degree of chlorination compared to the Orbitrap data, typically Cl_6_ for qToF and Cl_7_ for Orbitrap. These discrepancies may also be influenced by the different GC and MS method parameters, as well as the use of standards.^9^ Homologue pattern variability was observed in LC-based techniques as well, where APCI demonstrated greater sensitivity to lower chlorinated homologues compared to ESI.^33^ The discrepancy in results depicts the impact of ionization techniques and instrumental methodologies on PCA pattern measurements.^9,10^ Still, CPxplorer was proven to be consistent at reproducing patterns aligned with prior studies while remaining sensitive to instrument-specific homologue distributions.

Due to their low vapor pressure, PCAs-C_18–30_ are analyzed exclusively using LC-based instruments.^34^ The results obtained were comparable to those previously reported.^29–31^ The same PCA-C_18–30_ homologue pattern was observed across all instruments studied, with PCAs-C_22–24_ being the most abundant homologue group. The reconstituted PCA-C_18–30_ patterns were constructed by using PCA standard mixtures from Dr. Ehrenstorfer (Augsburg, Germany) along with several technical CP mixtures. However, the Dr. Ehrenstorfer standard mixtures were largely dominated by PCAs-C_18–20,_ ^35^ leading to reconstituted patterns primarily dominated by the CP technical mixtures Paroil CW 40, Witaclor 549, Uniclor40, and Hüls 40N. Indeed, the GoPF for the PCAs-C_18–30_ was very low when only the PCA mixtures from Dr. Ehrenstorfer were considered for deconvolution.^36^ When the technical mixtures Paroil CW 40 and Unichlor 40 were applied for quantification, the GoPF was 0.87 ± 0.02 for three replicates. CP technical mixtures are not composed purely of PCAs,^1^ which adds complexity to PCA analysis, since some interferences will not be resolved, mainly when using LRMS. This highlights the urgent need for longer-chain PCA standards,^12^ as currently no single chain congener standards are available for PCAs-C_≥18_.

Standards for PCAs-C_10–17_ at carbon chain length congener level are available, such as PCAs-C_10_ 52.5% Cl from Chiron.^37^ CPxplorer was also evaluated for PCA quantification using single-chain standards; see Figure 2 for the results acquired by GC-NCI-qToF. Additionally, PCAs-C_10–17_ in fish tissue material, ERM-CE100, were quantified using both PCA standard mixtures from Dr. Ehrenstorfer and the single-chain standards from Chiron. The deconvoluted pattern for PCAs-C_10–13_ was dominated by PCAs-C_13_, followed by PCAs-C_11_, PCAs-C_12_, and PCAs-C_10_ congeners, matching the pattern described by Ricci et al.^23^ Accordingly, 50.23% Cl for PCAs-C_13_ was the standard that contributed most to the CPquant reconstituted PCAs-C_10–13_ pattern. Overall, the GoPF between the deconvoluted and measured patterns matched >0.85 for all replicates. CPquant quantified the PCAs-C_10–13_ levels at 51 ± 9 μg/g and PCAs-C_14–17_ at 37 ± 9 μg/g, aligning with the certified values that were set at 22–40 and 27–61 μg/kg for PCAs-C_10–13_ and PCAs-C_14–17_, respectively.^23^ The measured concentration of PCAs-C_10–13_ was slightly higher than the certified range, but this deviation was considered acceptable in the context of known analytical variability and matrix complexity.

On the whole, CPxplorer was validated across a wide range of instruments, data formats, and standard mixtures, demonstrating its suitability for PCA data analysis and quantification. It was also shown to be a fast tool for exploring PCA congener and homologue patterns as well as a valuable resource for understanding the influence of instrumentation on PCA quantification.

#### 3.1.2 Results Comparability

The results obtained by CPxplorer were reproducible to those samples acquired with other mass spectral data treatment tools. For this, the potential mass spectral interferences of PCAs identified by CPions were further confirmed by CP-Hunter. CP-Hunter is a web-based platform that facilitates the data evaluation and assessment of single isotope clusters.^19^ CPions spotted the clusters of PCA and PCO homologues with the same carbon chain and chlorine atoms to interfere at resolution powers under 20,000, aligning with results obtained by CP-Hunter,^19^ and prior studies.^38,39^

CPions also identified potential interferences of BCAs and PCAs, which required a minimum resolution power of 20,000 to clarify the mass spectral signals matching the recommendation from Chibwe et al.^40^ To overcome this overlapping issue, McGrath et al.^18^ suggested the use of CP-seeker for the comparison of complete isotopologue pattern and *m*/*z* values of targeted ions with exact theoretical values. Here, CPxplorer described the same PCA and BCA patterns as CP-seeker for the same sample by applying a 20,000 mass resolution in CPions and excluding lower-intensity ions. It is crucial to emphasize that any exclusion of ions must strictly comply with all of the outlined criteria for data integration in Skyline.

The use of Skyline and the proposed criteria for peak selection were further validated by the comparability of the results obtained by CPxplorer to those acquired by the vendor software MassHunter and TraceFinder (Table 1 and Figure S5). All tools generated comparable integration results, with ∑PCAs-C_10–13_ levels consistent with those reported in previous studies for NIST-SRM-2585,^22,29–31^ Table 1. This suggests that the proposed data integration criteria are valid for PCA identification and quantification, representing a significant step toward harmonizing data processing and enhancing comparability across studies.

**Table 1:** Average Values of ∑PCAs-C_10‑13_ in μg/g in Two Replicates of NIST-2585 Dust^a^

instrument and ionization technique	software	∑PCAs-C_10–13_ (μg/g) by R script from Perkons et al.^20^	standard deviation	time spent for data processing
GC-NCI-Orbitrap	Trace Finder	5.20	3%	6 h
	Skyline	5.46		10 min
LC-ESI-qToF	Masshunter	9.32	0%	4 h
	Skyline	9.27		10 min

a The analysis was performed using GC-NCI-Orbitrap and LC-ESI-qToF, and the data were treated by TraceFinder/Skyline and MassHunter/Skyline, respectively. The quantification results were calculated using the script from Perkons et al.^20^ The time spent refers to the data treatment duration once the method was developed.

The peak integration data acquired from different software were quantified by the R script from Perkons et al.^20^ (see Table 1). These values were comparable to the quantification results obtained by CPquant for the same data: 6.3 and 8.8 μg/g for ∑PCAs-C_10–13_ when using GC-NCI-Orbitrap and LC-ESI-qToF, respectively. The small variation between the script from Perkons et al.^20^ and CPquant can be explained by the fact that the script from Perkons et al.^20^ performs deconvolution using the response from two standard mixtures at a time, while CPquant operates on the nnls package from R, which provides functions for the solution of linear systems by non-negative least-squares using all the standard mixtures available in the data set.

### 3.2 Application of CPxplorer in Environmental Analysis

To evaluate the robustness of CPxplorer in addressing matrix complexities, the performance of CPxplorer was tested across a range of environmental and biological matrices with varying degrees of complexity. These matrices were selected not only to reflect realistic analytical scenarios but also for their relevance to human exposure assessment, a particularly important aspect given the current lack of exposure limits and comprehensive risk assessments for PCAs.^41^ While CPxplorer is not limited to these types of samples, this evaluation aims to demonstrate its potential as a reliable tool in reducing interference-related challenges and contributing to the broader goal of improving exposure and risk assessment of PCAs. Moreover, a comprehensive understanding of human exposure to CP mixtures requires evaluating other halogenated compounds present alongside PCAs in technical mixtures, an aspect that was also assessed by using CPxplorer.

#### 3.2.1 Matrices Relevant for Human Exposure to PCAs: IOF, Indoor Dust, SWB, and Food

The current findings identified PCAs-C_14–17_ as the PCA group posing the highest exposure levels in the studied Swedish household, with ∑PCAs-C_14–17_ reaching 268.2 ng/m^2^ and 22.3 μg/g in IOF and indoor dust, respectively. Bai et al.^42^ also described an IOF pattern where PCAs-C_14–17_ were the most abundant PCA group, while Wong et al.^43^ measured higher levels of PCAs-C_14–17_ over PCAs-C_10–13_ in indoor Swedish dust as well. This PCA pattern dominated by PCAs-C_14–17_ was also seen in the SWB that was used to investigate the inhalation and dermal exposure to PCAs of a participant living in the sampled household (Figure 3A). Over a week, the SWB accumulated 0.9 μg/g (w/w), 5.5 μg/g (w/w), and 1.1 μg/g (w/w) of ∑PCAs-C_10–13_, ∑PCAs-C_14–17_, and ∑PCAs-C_18–30_, respectively. This results align with the ranges described by Yin et al.^44^ in SWBs that were worn by participants living in Belgium households.

**Figure 3:**
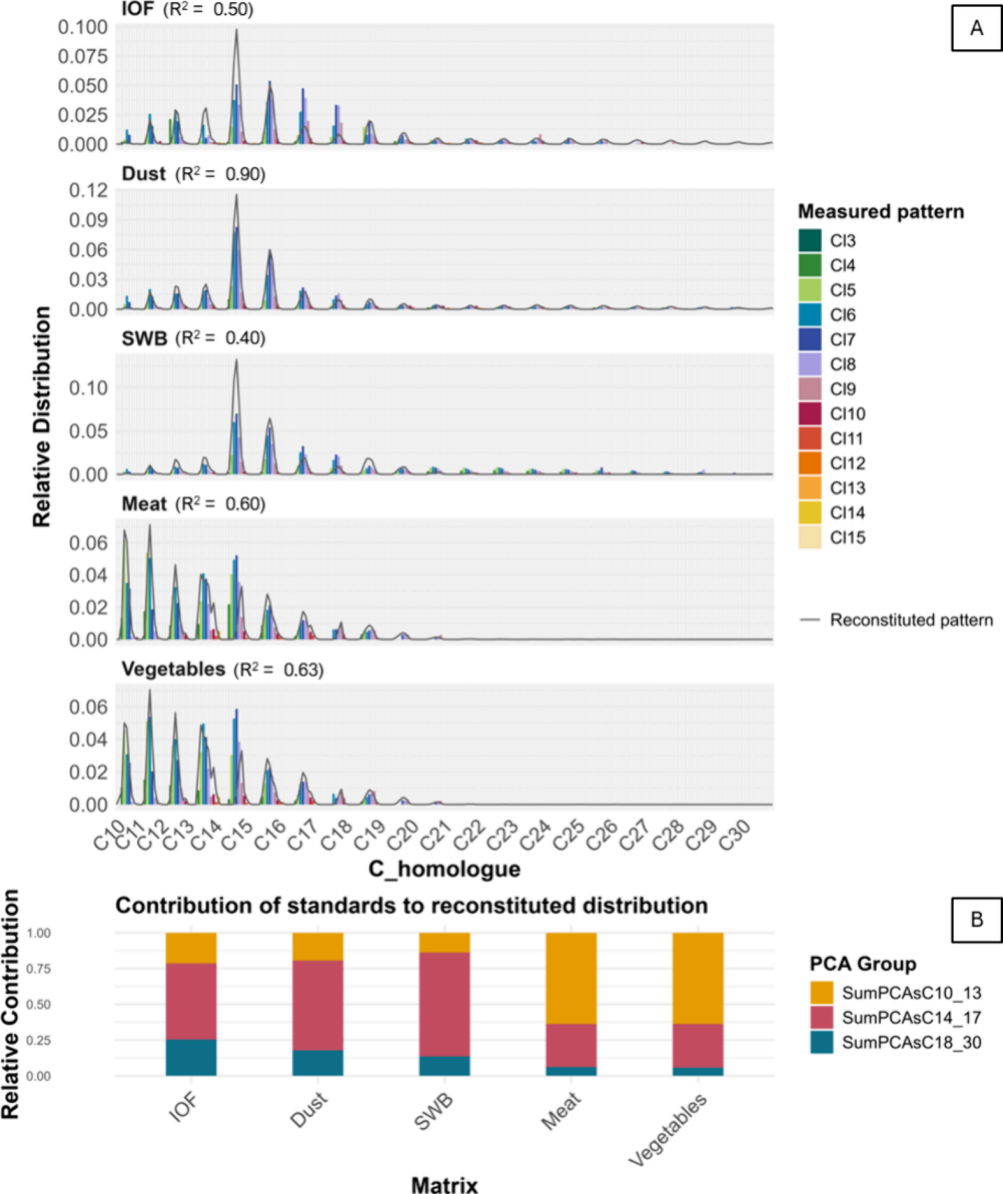
(A) PCA homologue group patterns in several matrices commonly used to assess human exposure, including IOF, indoor dust, SWB, meat, and vegetables. *R* ^2^ shows the GoPF between the measured and the reconstituted patterns. (B) The relative contribution of the standards to reconstitute the PCA homologue pattern in the sample and grouped into ∑PCAs-C_10–13_, ∑PCAs-C_14–17_, and ∑PCAs-C_18–30_, from top to bottom. Data were acquired from CPquant; the app also offers graphical displays equivalent to Figure 3, see Figure S4.

CPquant reconstituted the measured patterns in dust using several technical CP mixtures with an *R* ^2^ = 0.90 GoPF (Figure 3A). However, the GoPF value was lower for the reconstituted PCA patterns in the IOF and SWB at 0.50 and 0.40, respectively (Figure 3A). The lack of GoPF of the deconvoluted pattern was attributed to PCAs-C_21–30_, which were measured in the sample but not in the standards. Due to lab limitations, the SWB was quantified using the standard mixtures from Dr. Ehrenstorfer, which, as previously discussed, were seen to contain a poor contribution of PCAs-C_21–30_.^35^ Accordingly, the fitness between deconvoluted and observed PCA-C_10–17_ patterns in the SWB increased to 0.77 when PCAs-C_18–30_ were excluded from quantification. While the GoPF reflects the PCA homologue group match between the standards and the sample, the current results confirm the implementation of the nnls-based deconvolution algorithm from CPquant. This was demonstrated by the ability of CPquant to reliably reproduce measured PCA patterns under realistic conditions that account for sample matrix complexities.

The described capability of CPquant to provide the GoPF fit for the deconvoluted patterns offers a key advantage as it helps identify discrepancies between the standards and the sample. The app provides both *R* ^2^ values and a visual inspection tool (Figures 3B, S4), allowing users to assess how well the deconvoluted pattern matches the sample distribution. Bogdal et al.^11^ suggested that an *R* ^2^ value of 0.50 serves as a cutoff for meaningful quantification of PCAs-C_10–13_. They estimated that a *R* ^2^ of 0.50 could lead to a quantification error of approximately a factor of 4, comparable to differences between independent analytical methods.^11^ Brandsma et al.^38^ estimated that the difference between measured and expected concentrations was within a factor of 2 for GoPF values >0.5, while Zhou et al.^39^ reported a factor of 1.4 for total PCA concentrations under the same GoPF threshold. These findings support the use of this threshold as a pragmatic balance between accuracy and analytical feasibility, and thus reporting *R* ^2^ represents a significant factor in quality assurance. Another quality assurance feature of CPxplorer is the possibility to automatically correct the instrumental signal with an internal standard (IS) and calculate the IS-based recoveries.^45^ Many PCA analysts perform this task manually, whereas CPquant streamlines the process through automation.

Overall, CPxplorer proved useful in analyzing indoor dust and IOF patterns that also match those in the SWB. The ability of CPquant to provide quantified values is a major step in understanding exposure levels, aiding in risk reduction and setting exposure limits. Nevertheless, complementary analysis is needed to make the SWB a quantitative tool for assessing human exposure, including calculation of the sampling rate of the SWB and recording of the sampling conditions, such as humidity, temperature, direct contact with surfaces and materials, and more.

Food is another matrix that contributes to human exposure of PCAs through ingestion.^46^ Therefore, CPxplorer was applied to quantify PCAs in frequently consumed foods from the Swedish market. When comparing food items to indoor dust and IOF, a higher contribution of ∑PCAs-C_10–13_ and lower PCAs-C_18–30_ could be seen in both meat and vegetables, Figure 3A. These differences in PCA patterns may indicate different sources of contamination (e.g., plastic food packaging^47^) for the groceries compared to the indoor matrices. This discrepancy could be helpful to identify the pathways driving human exposure when studying PCA uptake in matrices, such as human serum.

CPquant quantified the levels of PCAs in food items with a GoPF > 0.60 (Figure 3A). The results showed that PCA levels in food were an order of magnitude lower than in indoor dust, with ∑PCAs-C_10–13_ at 19 ng/g (w/w) and 7.5 ng/g (w/w), ∑PCAs-C_14–17_ at 16 ng/g (w/w) and 5.9 ng/g (w/w), and ∑PCAs-C_18–30_ at 7.8 ng/g (w/w) and 4.0 ng/g (w/w) for meat and vegetables, respectively. As previously mentioned, the content of PCAs in food were close to the quantification limits, where the method detection limit (MDL) of ∑PCAs-C_10–13_ was 17 and 6.5 ng/g, of ∑PCAs-C_14–17_ was 14 and 5.4 ng/g, and of ∑PCAs-C_18–30_ was 5.9 and 2.3 ng/g for meat and vegetables, respectively. These results align with the levels in the literature,^48,49^ such as the concentrations reported by Ding et al.^50^ who measured in meat 7.6–78.1, 2.6–37.4, and 0.3–66.0 ng/g of ∑PCAs-C_10–13_, ∑PCAs-C_14–17_, and ∑PCAs-C_18–30_, respectively, and in vegetables <MDL–4.9, 1.4–50.7, and 0.2–199.5 ng/g of PCAs-C_10–13_, PCAs-C_14–17_, and PCAs-C_18–30_, respectively, providing a further confirmation to the CPxplorer workflow.

The quantification of Swedish food matrices, which have low PCA content and exhibit high blank contamination of PCAs-C_10–17_, is another showcase advantage of CPxplorer.^28,46^ CPquant enabled blank subtraction for each batch analysis. Additionally, CPxplorer proved highly effective for understanding blank contamination since CPquant applies the deconvolution method^11,20^ to quantify PCA levels in the blanks and the GoPF aids understanding if some homologue groups are not present in the blanks.^51^ For instance, the average ∑PCAs-C_10–30_ content in the blanks of the food samples batch was 10.2 ng/g, with an *R* ^2^ value of 0.67. The deviation from a perfect GoPF (*R* ^2^ = 1) was due to the absence of some homologue groups in the blanks compared to the standards. Complementarily, CPquant can also calculate method detection limit based on blank signals.

The swift data processing of CPxplorer enabled comprehensive analyses of multiple exposure sources, overcoming the challenges posed by the tedious nature of a large data set in some PCA investigations. Moreover, applying CPxplorer across a broad range of matrices demonstrated its capacity to overcome interference from other organic compounds, a key challenge in accurate PCA identification and quantification.^52^ By streamlining PCA quantification across diverse sample types, CPxplorer demonstrates its value as a practical and accessible tool for laboratories working with this complex contaminant group.

#### 3.2.2 PCA-Related Analogues

To gain a comprehensive understanding of human exposure to CP mixtures, it is essential to evaluate other halogenated compounds present in technical mixtures alongside PCAs.^18,40^ CPxplorer has demonstrated its effectiveness as a reliable tool for assessing the presence of PCOs in NIST-SRM-2585 indoor dust. The relative distribution based on the GC-NCI-Orbitrap instrumental signals revealed the highest presence of PCAs and PCOs with C_12_Cl_7_, as depicted in Figure S6. Both PCA and PCO patterns were predominantly composed of compounds containing C_11_–C_14_ and Cl_6_–Cl_8_. Notably, PCOs-Cl_6_ were detected more frequently than PCAs-Cl_6_, while PCOs with nine chlorines were less common than their PCA equivalents, particularly for those containing 11 and 12 carbons. Contrarily, the PCA/PCO pattern measured in NIST-SRM-2585 by LC-APCI-Orbitrap did not show significant discrepancies in chlorination patterns for PCAs-C_10–17_ and PCOs-C_10–17_ (Figure S7).

The capability of CPxplorer to accurately differentiate between interfering PCA and PCO ions helps to minimize the risk of “false positives”, as mentioned by Mendo Diaz et al.^19^ This issue arises from the near-complete overlap of isotopic clusters of PCA and PCO homologues with identical carbon chains and chlorine atoms^33,52^ (Figure 4). CPxplorer can partly overcome this challenge by removing problematic *m*/*z* values in CPions from the target list and ensuring that these ions are not considered for PCA identification in Skyline. This process was further validated by confirming well-defined profile MS data, isotopic distributions consistent with the standard, and adherence to strict mass error criteria. Additionally, our results were in good agreement with CP-Seeker,^18^ which identifies the interferences by comparing the whole experimental and theoretical isotopic patterns.

**Figure 4:**
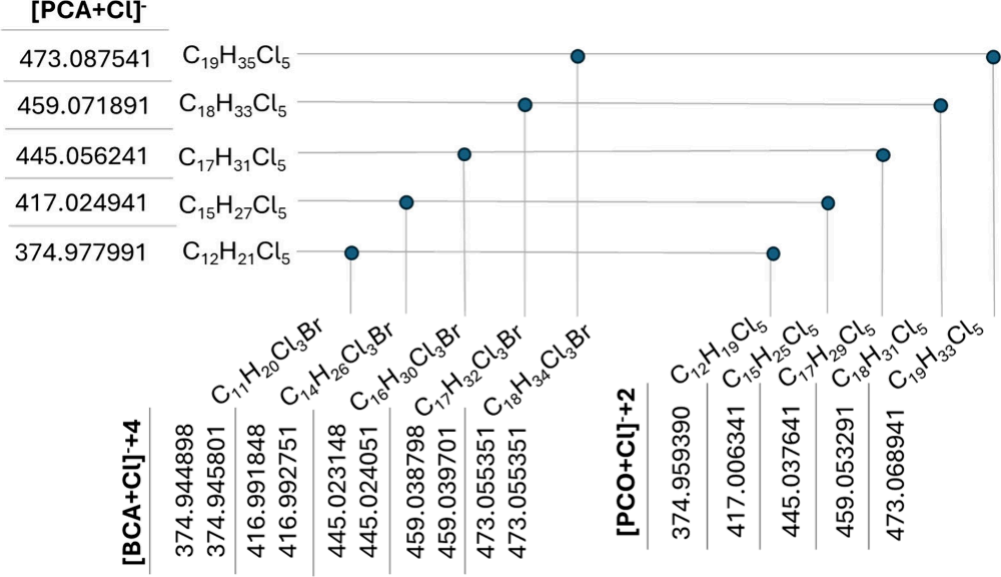
Selected interferences of [M + Cl]^−^ adducts of PCA-Cl_5_, BCAs, and PCOs calculated by CPions. The axis shows the parent formula and the *m*/*z* values of the interfering ions, and the dots refer the ions that overlap with each other at 20 000 resolution power. The [PCA + Cl]^−^ ions correspond to the ^35^Cl_6_ isotopologues, the [BCA + Cl]^−^+4 ions correspond to the ^35^Cl_2_^37^Cl_2_^79^Br and ^35^Cl_3_^37^Cl^81^Br isotopologues, and the [PCO + Cl]^−^+2 ions correspond to the ^35^Cl_5_^37^Cl isotopologues. Note: The LC-qToF used for data acquisition had a resolving power of 20 000; therefore, interferences were assessed at this resolution. General molecular formulas and examples of BCAs and PCOs can be found in Table S4.

Although PCAs can be distinguished from some PCOs, potential interferences must be considered. Specifically, in chlorine-enhanced methods where the in-source fragment [PCA + Cl–HCl]^−^ shares the same chemical formula as the [PCO + Cl]^−^ adduct of the PCO containing one chlorine less.^53^ Conversely, when targeting [M – Cl]^−^ and [M – H]^−^ fragments, the [PCA – H – HCl]^−^ fragment has the exact same *m*/*z* value as [PCO – Cl]^−^ of the PCO containing the same number of carbons and chlorines. Given the wide retention time ranges and variable fragmentation efficiencies of numerous PCA and PCO congeners,^54^ their peaks often differ significantly in shape. Therefore, distinguishing PCA in-source fragments from PCO-related ions based on peak shape or alignment with other PCA fragments/adducts is unreliable.^53^ CPxplorer is flexible for peak integration, so the user can account for the described potential interference and remove or reintegrate the peaks in Skyline if needed.

Moreover, PCAs are also found in other technical mixtures containing different polyhalogenated alkanes (PXAs).^40^ The presence of PXAs also hinders the mass spectral separation and signal assignment of PCAs.^18^ Indeed, BCAs that have one carbon less, two chlorines fewer, and one bromine more than PCAs lead to “false positives” (Figure 4). The CPions feature enabled the identification of potential “false positives” arising from BCAs and ensured their exclusion from the ion target list. This facilitated the accurate identification of PCAs and confirmed the absence of BCAs in the analyzed samples, which were also investigated in the dust samples but not detected.

To gain a deeper understanding of the fate of PCAs, it is crucial to also investigate their transformation products; see Table S4 for the general molecular formula and examples of PCA transformation products. CPxplorer was able to identify some of the phase I metabolites reported by Chen et al.^25^ in rice plant roots (*Oryza sativa* Japonica cv. Nipponbare) previously exposed to 1,2,5,6,9,10-C_10_H_16_Cl_6_. The metabolites included C_10_Cl_5_ and C_10_Cl_6_ hydroxy-PCAs, C_10_Cl_5_ and C_10_Cl_6_ oxo-PCAs, C_10_Cl_6_ PCAs-H + SO_4_H, and C_10_Cl_4_, C_10_Cl_5_, C_11_Cl_5_, C_12_Cl_5_, and C_12_Cl_6_ carboxy-PCAs. These results highlighted the need for efficient chromatographic separation as some oxo-PCAs, carboxyl-PCAs, and acetyl-PCAs share an identical molecular formula, making differentiation by only mass spectrometry impossible. For instance, as shown in Figure S8, the carboxy-PCA-C_12_Cl_5_O_2_ extracted ion chromatogram suggests that more than one compound is coeluting. However, mass spectral data alone cannot clarify this, emphasizing the critical need for manual data verification. Skyline has an advantage that it enables users to empirically adjust, exclude, and correct automated data integration.

### 3.3 Environmental Implications

The widespread presence of PCAs across a broad range of environmental matrices and their PBT properties are of significant concern. Industrial production of CP mixtures has been ongoing since the 1930s, with some estimates on current global output exceeding 1 million tonnes per year.^3,6^ However, these contaminants remain poorly understood and their analysis continues to be challenging. CPxplorer aims to bridge this gap by simplifying CP mixture data analysis and fostering harmonization across laboratories. This will encourage more laboratories to quantify PCAs in environmental matrices, leading to more consistent results and improving risk assessment.

As an open source and freely available tool, CPxplorer features user-friendly interactive GUIs (Figures S2–S4) with comprehensive manuals, ensuring accessibility and ease of use. This is particularly important since PCAs-C_10–13_ and PCAs-C_14–17_ have been added to the Stockholm Convention.^5^ As these compounds are globally regulated, more laboratories will need to implement PCA analysis, a process that is complex and time-consuming. Therefore, the intuitive design of CPxplorer makes it easy to adopt this workflow, enhancing the efficiency of PCA analysis implementation for research and commercial laboratories.

In future developments, CPxplorer can also be used for the quantification of BCAs, PCOs, and other byproducts present in CP mixtures and environmental samples when standards become available.^18,55^ CPxplorer is a customizable framework that makes new implementation feasible, such as more QA/QC features and multivariate analysis. Additionally, it allows for the inclusion of additional adduct/fragment ions upon request, and the code source is openly available the GitHub repository: https://github.com/WBS-TW/CPxplorer.

## Supplementary Material



## Notes

Detailed description of sample preparation and analysis of NIST-SRM-2585, ERM-CE100, IOF, indoor dust, SWB, meat and vegetables, and rice plant exposure; overview table of sample preparation; identification of mass spectral interferences and target ion list generation using CPions; signal elucidation and peak integration using Skyline; deconvolution and quantification of homologue groups using CPquant; quantification approach used by CPquant; quantification results for NIST-SRM-2585 and matrices used to address human exposure; overview on the formulas and ions of polychlorinated alkanes (PCAs), transformation products derived from PCAs, polychlorinated olefins (PCOs), and bromochloro alkanes (BCAs); GUI display of interfering ion results from CPions, Skyline analysis, and CPquant quantification; relative distribution of PCAs-C_10–17_ and PCOs-C_10–17_ measured in NIST-SRM-2585; extracted ion chromatogram of C_12_H_19_Cl_5_O_2_ [M + Cl]^−^; peak area for PCA-C_10–13_ homologue groups from two replicates of NIST-SRM-2585 indoor dust (PDF)
