# Supplementary material for: Streamlining Quantification and Data Harmonization of Polychlorinated Alkanes Using a Platform-Independent Workflow
Source: Environ Sci Technol. 2025 Oct 9;59(41):22074–84. doi: 10.1021/acs.est.5c04928 (PMC12550811; doi:10.1021/acs.est.5c04928)
Supplement: Supplementary file 1 [file es5c04928_si_001.pdf]

# SUPPORTING INFORMATION

# Streamlining Quantification and Data Harmonization of Polychlorinated Alkanes Using a Platform-Independent Workflow


**Idoia Beloki Ezker[1], Bo Yuan[2], Anders Røsrud Borgen[3], Jiyan Liu[4,5], Yawei Wang[4], Thanh Wang[1,6]**

**[1] Department of Physics, Chemistry and Biology (IFM), Linköping University, 581 83 Linköping, Sweden**

**[2] Department of Chemistry, Norwegian University of Science and Technology, 7491 Trondheim, Norway**

**[3] Environmental Chemistry and Health Effects, NILU, 2007 Kjeller, Norway**

**[4] State Key Laboratory of Environmental Chemistry and Ecotoxicology, Research Center for Eco-Environmental Sciences, Chinese Academy of Sciences, Beijing, 100085, China**

**[5] School of Environment, Hangzhou Institute for Advanced Study, University of Chinese Academy of Sciences, Hangzhou, Zhejiang, 310000, China**

**[6] Department of Thematic Studies – Environmental Change, Linköping University, 581 83 Linköping, Sweden**

**E-mail contact:** thanh.wang@liu.se

## Table of contents

*Section S1: Sample preparation and analysis of NIST-SRM-2585* ............................... *S3*

*Section S2: Sample preparation and analysis of ERM-CE100* ..................................... *S3*

*Section S3: Sample preparation and analysis of IOF* ................................................. *S3*

*Section S4: Sample preparation and analysis of indoor dust* ..................................... *S4*

*Section S5: Sample preparation and analysis of SWB* ............................................... *S4*

*Section S6: Sample preparation and analysis of meat and vegetables* ........................ *S5*

*Section S7: Sample preparation and analysis of Rice plant exposure* .......................... *S6*

*Table S1: Sample preparation overview* ...................................................................... *S7*

*Section S8: CPions: Identification of Mass Spectral Interferences and Target Ion List Generation* ........................................................................................................ *S8*

*Section S9: Skyline: Signal elucidation and peak integration* ........................................... *S9*

*Section S10: CPquant: Deconvolution and Quantification of Homologue Groups* ........... *S10*

*Figure S1: Quantification approach used by CPquant* .................................................... *S12*

*Table S2: Quantification results for the NIST-SRM-2585* .................................... .......... *S13*

*Table S3: Quantification results for the matrices used to address human exposure* ........ *S13*

*Table S4: Overview of polychlorinated alkanes (PCAs), transformation products derived from PCAs, polychlorinated olefins (PCOs) and bromochloro alkanes (BCAs* .......................... *S14*

*Figure S2: Interfering ion results GUI display from CPions.* .......................................... *S15*

*Figure S3: Skyline GUI display for PCA data analysis* ................................................... *S16*

*Figure S4: CPquant GUI display for PCA quantification.* .......................................... *S17*

*Figure S5: Peak area for PCA-C10-13 homologue groups from two replicated of NIST-SRM-2585 indoor dust* ........................................................................................ *S18*

*Figure S6: Relative distribution of (A) PCAs-$C_{10-17}$ and (B) PCOs-$C_{10-17}$ measured in the NIST-SRM-2585* ......................................................................................... *S19*

*Figure S7: Relative distribution of (A) PCAs-$C_{10-17}$ and (B) PCOs-$C_{10-17}$ measured in the NIST-SRM-2585* ......................................................................................... *S20*

*Figure S8: Extracted ion chromatogram of $C_{12}H_{19}Cl_5O_2$ $[M + Cl]^-$* .................................... *S20*

*References* ................................................................................................... *S21*

### *Section S1: Sample preparation and analysis of NIST-SRM-2585*

NIST-SRM-2585 indoor dust reported in the current study was extracted and analyzed by different laboratories including our own. The NIST-SRM-2585 analyzed by liquid chromatography (LC)-electrospray ionization (ESI)-quadrupole time of flight (qToF) and gas chromatography- negative chemical ionization (NCI)-Orbitrap were pretreated and analyzed as described in Section S4. The only variation was that for the GC-NCI-Orbitrap the $[M - Cl]^-$ were monitored for the PCAs-$C_{10-17}$. The data acquired by LC- atmospheric pressure chemical ionization (APCI)- Orbitrap, and direct injection (DI)- APCI-Orbitrap was pretreated and analyzed as described by Spaan et al. (2023).[1] Finally, the NIST-SRM-2585 analyzed by GC-electron capture negative ionization (ECNI)-qToF were extracted by three cycles of 1.5 mL of dichloromethane DCM: hexane (1:1, v/v) mixture in sonication bath, and cleaned-up using a multilayer column packed from bottom to top with 2 g of silica (deactivated with 2.5% H2O), 6 g of 44% sulfuric acid silica, and 4 g of anhydrous sodium sulfate. For quantification of the NIST-SRM-2585 (GC-ECNI-qToF) the single chain and technical mixture standards described in Section S6 were used. For all NIST-SRM-2585 $^{13}C_{10}$- 1,1,1,3,10,12,12,12-octachlorododecane was used as internal standard and CPxplorer was used for data quantification.

### *Section S2: Sample preparation and analysis of ERM-CE100*

The ERM-CE100 samples were pretreated as described by Ricci et al. (2024).[2] Briefly, 5 g of the fish oil was spiked by $^{13}C_{10}$- 1,1,1,3,10,12,12,12- octachlorododecane and extracted by cold column extraction using 100 mL of cyclohexane:ethyl acetate 1:1. Clean-up on 10 g of 44 % $H_2SO_4$ silica and fractionation on 4 g 1.5 % deactivated Florisil. Addition of tetrachloronapthalene as a syringe standard. For the analysis GC-ECNI-HRMS (qToF) was used where the $[M - Cl]^-$ ions were monitored. The following single chain mixtures were used for quantification in CPxplorer were purchased from Chiron AS: $C_{10}$: 50.18, 65.02 %Cl; $C_{11}$: 50.21, 65.25 %Cl; $C_{12}$: 50.18, 65.08 %Cl; $C_{13}$: 50.23, 65.18 %Cl; and $C_{14}$ 52%Cl; as well as the CP mixtures: $C_{10\text{-}C13}$ 51.5 %Cl, $C_{10-13}$ 55.5 %Cl, $C_{10-13}$ 63 %Cl; $C_{14-17}$ 42 %Cl, $C_{14-17}$ 52 %Cl, $C_{14\text{-}C17}$ 57 %Cl.

### *Section S3: Sample preparation and analysis of IOF*

Indoor organic film (IOF) samples were collected in Sweden during May 2023. Kleenex were used as sampling tools and they were precleaned by sonication in DCM:Hex, 3:1, for 15 minutes, and dried under $N_2$ flow in a closed and cleaned environment under the fume hood. For sampling the IOF, the Kleenex were wetted by 3 mL isopropanol and 1 $m^2$ of the glass windows were wiped. In the lab, the entire Kleenex was then cut into small pieces, spiked with 20 ng of $^{13}C_{10}$-hexachlorodecane, and extracted by three sonication cycles of 15 minutes

in 6 mL DCM:Hex (3:1). The supernatants were collected and combined and dried to 1 mL under $N_2$ flow prior to the clean-up step. Extracts were then cleaned in glass columns packed with 0.5 g each of silica gel, Florisil, and sodium sulfate, from bottom to top, eluted with 15 mL of DCM: hexane (1:1, v/v), concentrated to 0.5 mL under $N_2$, and spiked with 20 ng of ε-hexachlorocyclohexane before analysis. The analysis was done by the Agilent 6546 LC-qToF in electrospray ionization (ESI), and $[M + Cl]^-$ ions were generated by the addition of 0.05 mM tetramethylammonium chloride into the mobile phases. The quantification was performed in CPxplorer using PCA standard mixtures from Dr. Ehrenstorfer (Augsburg, Germany) containing PCAs-$C_{10-13}$ with 51.5%, 55.5%, and 63% chlorine content, PCAs-$C_{14-17}$ with 42%, 52%, and 57% chlorine content, and PCAs-$C_{18-30}$ 36% and 49%. Complementarily, the technical mixtures Uniclor40 from Neville Chemical Co (USA), and Paroil CW 40 from Dover Chemical Corporation (USA) were also included.

### *Section S4: Sample preparation and analysis of indoor dust*

Indoor dust samples were collected in Sweden during May 2023. Indoor dust (0.05 ± 0.003 g) was transferred to 10 mL glass test tubes and spiked with 20 ng of $^{13}C_{10}$-hexachlorodecane. Samples were extracted with 1.5 mL of dichloromethane DCM: hexane (3:1, v/v), vortexed for 10 seconds, sonicated for 15 minutes, then centrifuged for 8 minutes at 3000 rpm. The supernatant was collected after three extraction cycles, combined, and concentrated to 1 mL under $N_2$ stream. Extracts were cleaned using columns with 0.5 g each of silica gel, Florisil, and sodium sulfate, eluted with 15 mL of DCM: hexane (1:1, v/v), concentrated to 0.5 mL, and spiked with 20 ng of $^{13}C_{12}$-octachlorododecane before analysis. Samples were analyzed using Agilent 6546 LC-qToF in electrospray ionization (ESI), and $[M+Cl]^-$ ions were monitored by adding 0.05 mM of tetramethylammonium chloride to the mobile phases. The quantification was performed in CPxplorer using PCA standard mixtures from Dr. Ehrenstorfer (Augsburg, Germany) containing PCAs-$C_{10-13}$ with 51.5%, 55.5%, and 63% chlorine content, PCAs-$C_{14-17}$ with 42%, 52%, and 57% chlorine content, and PCAs-$C_{18-30}$ 36% and 49%. Complementarily, the technical mixtures Uniclor40 from Neville Chemical Co (USA), and Paroil CW 40 from Dover Chemical Corporation (USA) were also included.

### *Section S5: Sample preparation and analysis of SWB*

Volunteers wore pre-cleaned silicone wristband (SWB) during seven days in June 2024. After that period, the SWB were covered with aluminum film and stored in sealed aluminum bags at -20°C until sample preparation. Prior to extraction, SWBs were cut into small pieces, homogenized, and 1 g was transferred to a previously burned 10 mL glass test tube. The samples were extracted by three cycles of sonication with 6.5 mL of toluene: acetone: hexane (1:3:9). The supernatants were collected and evaporated under $N_2$ until 1 mL. The

combined extracts were cleaned using a glass column with silica, Florisil, and sodium sulfate. The multi-layer was activated with Hexane, and the samples were eluted with Hex and the mixture Hex:DCM (1:1). The samples were collected in a clean vial. The samples were then concentrated to dryness under $N_2$. Finally, samples were redissolved with acetonitrile and 20 ng of $^{13}C_{12}$-octachlorododecane were spiked. The samples were analysed by LC-qtof (Agilent 1290 Infinity LC, Agilent 6550 iFunnel HRMS) with ESI in negative mode. For quantifying PCAs-$C_{10-30}$, the [M + Cl]- ions were used, generated by adding 0.05 mM of tetramethylammonium chloride to the mobile phases. The quantification was performed in CPxplorer using PCA standard mixtures from Dr. Ehrenstorfer (Augsburg, Germany) containing PCAs-$C_{10-13}$ with 51.5%, 55.5%, and 63% chlorine content, PCAs-$C_{14-17}$ with 42%, 52%, and 57% chlorine content, and PCAs-$C_{18-30}$ 36% and 49%.

***Section S6: Sample preparation and analysis of meat and vegetables***

The sample preparation method was adapted from previous studies.[3] Briefly, a mass equivalent to 0.3 g of fat was weighed and spiked with 10 ng of the internal standard, $^{13}C_{10}$-1,5,5,6,6,10-hexachlorodecane. The samples were homogenized for two cycles of 30 seconds with 14 mL of hexane:acetone (14:35) and then subjected to two solid-liquid extraction cycles with 10 mL of hexane:diethyl ether (9:1). The supernatants from the solid and liquid dairy samples were dehydrated with 10 mL of aqueous 0.9% sodium chloride and 0.1 M phosphoric acid, followed by two liquid-liquid extraction cycles with 5 mL of hexane. Clean-up was performed using a multilayer column packed from bottom to top with 2 g of silica (deactivated with 2.5% $H_2O$), 6 g of 44% sulfuric acid silica, and 4 g of anhydrous sodium sulfate. The concentrated extracts were loaded onto the column and eluted with 15 mL of hexane:diethyl ether (1:1, v/v). The eluent was concentrated, and 10 ng of $^{13}C_{12}$-1,1,1,3,10,12,12-octachlorododecane was added as a recovery standard. The extracts were then solvent exchanged to acetonitrile before instrumental analysis. PCAs were measured using liquid chromatography coupled with quadrupole time-of-flight mass spectrometer (LC-qToF, Agilent 6546) in electrospray negative ionization mode. For quantifying PCAs-$C_{10-30}$, the [M + Cl]- ions were used, generated by adding 0.05 mM of tetramethylammonium chloride to the mobile phases. CPxplorer was used for the pattern-deconvolution algorithm[4,5] of PCA-$C_{10-13}$ and PCA-$C_{14-17}$, single-chain standards (Chiron AS) for $C_{10}$ (52.5% and 58.4% Cl), $C_{11}$ (52.3% and 57.7% Cl), $C_{12}$ (53.8% and 57.3% Cl), $C_{13}$ (45.9% and 60% Cl), $C_{14}$ (49.2% and 58.7% Cl), $C_{15}$ (47.7% and 59.3% Cl), C16 (51.5% and 58.4% Cl), and $C_{17}$ (56.3% Cl) were used. For PCA-$C_{18-30}$ quantification, standard mixtures from Dr. Ehrenstorfer (Augsburg, Germany) containing 36.0% Cl and 49.0% Cl, and technical mixtures Uniclor40 from Neville Chemical Co (USA) and Paroil CW 40 from Dover Chemical Corporation (USA) were used.

### *Section S7: Sample preparation and analysis of rice plant exposure*

The rice roots were sampled, freeze-dried, and stored at -20°C for later analysis. The sample preparation involved collecting the roots from rice seedlings exposed to root exudates or cultivation solutions. Detailed pretreatment procedures and instrumental settings for analysis are provided in Chen et al. (2022).[6] The data was treated by the modules CPions and Skyline of CPxplorer, and compared with CPseeker [7].

***Table S1:*** **Sample preparation overview, detailed information in Sections S1-7**

| Section | Matrix | Extraction & Clean-up | Instrumentation and Ionization | Standards Used |
|---|---|---|---|---|
| S1 | NIST SRM-2585 (Indoor Dust) | Varies by lab: - GC-ECNI-qToF: 3x 1.5 mL DCM:Hex (1:1), multilayer silica/sulfuric/sodium sulfate | - LC-ESI-qToF - LC-APCI-Orbitrap - DI-APCI-Orbitrap - GC-NCI-Orbitrap ($[M-Cl]^-$) - GC-ECNI-qToF | Dr. Ehrenstorfer $C_{10–13}$, $C_{14–17}$, $C_{18–30}$; $C_{10}$–$C_{14}$ single chain |
| S2 | ERM-CE100 (Fish Oil) | Cold column extraction (100 mL cyclohexane:ethyl acetate 1:1), sulfuric acid silica & Florisil clean-up | GC-ECNI-qToF, monitoring $[M-Cl]^-$ | Chiron As $C_{10}$–$C_{14}$ single chain; $C_{10–13}$ & $C_{14–17}$ mixtures |
| S3 | Indoor Organic Film (IOF) | Pre-cleaned Kleenex wiped over 1 $m^2$ window, extracted 3x with 6 mL DCM:Hex (3:1), cleaned on silica/Florisil/sodium sulfate | LC-qToF (ESI), $[M+Cl]^-$ generated with TMA-Cl | Dr. Ehrenstorfer $C_{10–13}$, $C_{14–17}$, $C_{18–30}$; Uniclor40 & Paroil CW 40 |
| S4 | Indoor Dust (Field Sample) | 3x extraction with 1.5 mL DCM:Hex (3:1), cleaned on silica/Florisil/sodium sulfate | LC-qToF (ESI), $[M+Cl]^-$ with TMA-Cl | Same as S3 |
| S5 | Silicone Wristbands (SWB) | 3x sonication with toluene:acetone:hexane (1:3:9), cleaned on silica/Florisil/sodium sulfate, eluted with Hex/Hex:DCM | LC-qToF (ESI), $[M+Cl]^-$ with TMA-Cl | Same as S3 |
| S6 | Meat & Vegetables | Homogenization in hexane:acetone, extraction in hexane:diethyl ether, aqueous phase dehydration, multilayer silica/sulfuric/sodium sulfate clean-up | LC-qToF (ESI), $[M+Cl]^-$ with TMA-Cl | $C_{10}$–$C_{14}$ single chain; Uniclor40 & Paroil CW 40 |
| S7 | Rice Roots (Exposure Study) | Freeze-dried roots; extraction & clean-up per Chen et al. (2022) | As per Chen et al. (2022); CPions & Skyline used | No quantification was performed |

## ***Section S8: CPions: Identification of Mass Spectral Interferences and Target Ion List Generation***

CPions is a core module of CPxplorer designed to support the selection of non-interfered m/z ions for polychlorinated alkane (PCA) quantification. Its primary purpose is to generate a refined target ion list by identifying potential spectral interferences, thereby improving accuracy and reliability in complex samples. The module operates by simulating theoretical isotopic patterns using enviPat,[8] and then assists in evaluating which ions are expected to overlap at the instrument's resolving power.

For start, the user defines key input parameters that determine which molecular formulas will be considered in the simulation. These include:

- The range of carbon atoms (C), chlorine atoms (Cl), and bromine atoms (Br) to include (e.g., $C_3$–$C_{40}$, $Cl_1$–$Cl_{15}$, $Br_1$–$Br_{15}$).
- The compound families to consider, including PCAs, polychlorinated olefins (PCOs), and brominated chlorinated alkanes (BCAs) in standard mode tab, with the option to also include transformation products (e.g., hydroxylated or unsaturated species) in the advanced mode tab.
- The adducts or fragments to monitor, such as $[PCA + Cl]^-$, $[PCA - Cl]^-$, $[PCA - H]^-$, and others depending on the ionization mode and analytical setup.
- The specific internal and recovery standards used in the analysis, including isotopically labeled compounds, which are automatically included in the interference evaluation workflow.

Once the molecular input space is defined, CPions simulates the isotopic patterns for each possible species and calculates whether two m/z values are distinguishable at the instrument's specified resolving power. These are defined as interfering ions if equation (1) is fulfilled:

$$Instrumental\ resolution < \frac{m/z}{\Delta\, m/z} \qquad (1)$$

For example, to distinguish between ions at m/z 234.5467 and 234.5461, a resolving power of 39,091 is required. If the data were acquired at a resolving power of 20,000, CPions would flag these ions as potentially unresolved and, therefore, subject to interference.

To further refine the target list, CPions allows the user to apply a relative abundance threshold, commonly set to 20% of the base peak. Only isotopologues exceeding this threshold are retained, ensuring that the most intense and relevant signals (which are more likely to not be affected by co-elution) are appropriately screened. Note that increasing the

threshold reduces the number of ions included in the target list; if set too high, only the base peak may be retained. The base peak is defined as the “quan” ion and the other isotopologues from the same homologue group are labeled as “qual”. However, this setting is optional; users can also export all ions to Excel for manual review and selection.

Briefly, CPions provides flexibility in how interferences are addressed:

- It automatically flags ions expected to overlap with others at the defined resolution, accounting not only for the selected PCA congeners but also for related compounds such as PCOs, BCAs, and internal standards.
- While CPions does not eliminate spectral interferences from the sample itself, it allows users to exclude potentially interfered ions from the target list, so these ions are not used for chromatographic peak identification or quantification.
- Alternatively, users may choose to manually review all candidate ions and select preferred quantifier/qualifier ions based on their own criteria.
- The resulting refined target list can then be exported directly to Skyline for chromatographic peak integration.

By identifying potential interferences early, either before data acquisition or during method development, CPions supports more reliable quantification and enables informed decisions about ion selection and acquisition strategies. This is particularly valuable when working with complex technical mixtures and diverse environmental or biological matrices.

More detailed guidance on parameter settings, customization options, and complete workflows is provided in the user manual and video tutorials, available in the CPxplorer GitHub repository.

### *Section S9: Skyline: Signal elucidation and peak integration*

The refined target list generated in CPions can be directly imported into Skyline for chromatographic peak identification and integration. One of the key strengths of this workflow is that instrumental data does not require format conversion, Skyline supports direct processing of raw files from most high-resolution mass spectrometry platforms, including .d, .wiff, .qgd, .raw, and .mzml file extensions from the major vendors Agilent and Bruker, Sciex, Shimadzu, Thermo and Waters.

CPxplorer proposes a set of standardized criteria for peak selection and integration within Skyline to ensure consistency and reliability across laboratories and instruments (Figure S3):

- Same retention time range for both the standard and the sample: Target peaks must fall within the same RT window, indicating consistent chromatographic behavior.

- Matching isotopic distribution between the standard and the sample: Instead of comparing to theoretical isotopic patterns, CPxplorer recommends comparing the measured isotopic pattern of the sample directly with that of the corresponding standard. This approach accounts for potential differences in ionization efficiencies across isotopologues, which may vary depending on the instrument or acquisition conditions, but are expected to remain consistent between the standard and the sample.
- MS profile data exhibiting a Gaussian distribution: In high-resolution MS data acquired in profile mode, ions are represented as continuous curves rather than discrete centroids. A Gaussian-shaped distribution of the ion signal across the m/z axis reflects proper resolution and stable ion transmission. This shape is essential to ensure accurate m/z determination, minimize integration errors, and avoid misinterpretation of overlapping or poorly resolved signals.
- Consistent peak shape across all monitored isotopologues, including both quantifier ("quan") and qualifier ("qual") ions: This supports the assumption that all monitored fragments originate from the same compound and co-elute under the same chromatographic conditions.
- Mass accuracy criteria: For Orbitrap instruments, the observed m/z values should fall within ±5 ppm of the expected value, while for qTOF instruments, a tolerance of ±15 ppm is applied.

Skyline provides a multipanel display where the described parameters, including retention time, mass error, isotopic distribution, and peak shape, can be evaluated simultaneously. If a peak is incorrectly integrated in one sample, discrepancies will typically appear in these other parameters (e.g., increased mass error or mismatched isotopic pattern), making issues easy to detect. The user can then manually reintegrate only the affected sample if needed. Additionally, Skyline supports synchronized integration, allowing the user to correct for multiple files at once when they fail to meet the criteria, without having to manually inspect each peak individually.

These criteria, when used in combination, enhance the robustness of the quantification and reduce the likelihood of integrating false positives or interferences. Full details and visual guidance on using Skyline with CPxplorer’s outputs are provided in the accompanying user manual and video tutorials, available through the GitHub repository.

### *Section S10: CPquant: Deconvolution and Quantification of Homologue Groups*

CPquant operates on the peak area results exported from Skyline and focuses exclusively on the quantifier (“quan”) ions selected during the target list generation. As illustrated in Figure S1, CPquant first determines the response factor for each homologue group in each

calibration standard, based on their respective calibration curves. The user can remove homologue groups from the standard if their calibration curve has an $R^2$ (goodness of calibration fit, GoCF) value below a user-defined threshold. CPquant then applies a non-negative least squares (nnls) algorithm to estimate the contribution of each standard mixture, optimizing the fit to match the relative abundances observed in the sample. These contributions are used to calculate the concentration of each homologue group in the sample.

From this, a deconvoluted pattern is constructed based on the calculated concentrations, while the measured pattern reflects the relative distribution derived directly from the integrated peak areas. The goodness of pattern fit (GoPF) using the coefficient of determination ($R^2$) value quantifies the agreement between these two patterns, with a GoPF of 1.0 indicating a perfect match (100%) and lower values reflecting decreasing similarity. The CPxplorer workflow adopts the $R^2$ threshold of 0.50 proposed by Bogdal et al. (2015),[4] based on their comprehensive sensitivity analysis described in the Supporting Information of that study.

Comprehensive instructions and visual guides for using CPquant, including features like blank signal subtraction, data normalization, and more, are available in the user manual and video tutorials on the GitHub repository.

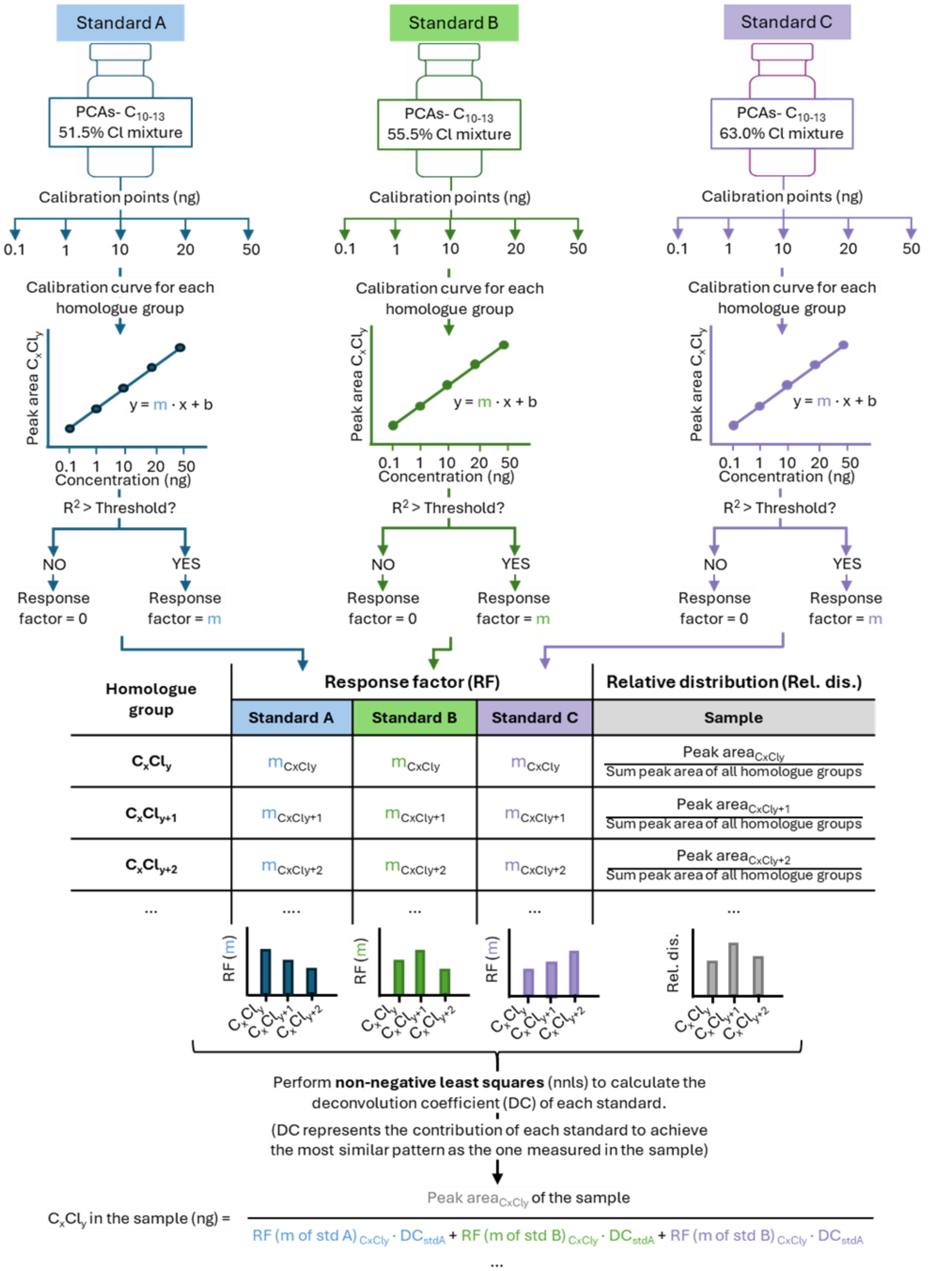


***Figure S1:*** Quantification approach used by CPquant to determine the concentration of

each homologue group in the sample. CPquant implements the method originally proposed by Bogdal et al. (2015),[4] incorporating the response factor calculation for each homologue group from Perkons et al. (2019),[5] and applies a non-negative least squares (NNLS) algorithm to solve for the contributions of the standard mixtures. Note (I): The standards and concentrations displayed in the figure are examples, CPquant is not restricted to them. Note (II): The $R^2$ refers to the GoCF and the threshold is set by the user.

***Table S2:*** Quantification results for the NIST-SRM-2585 obtained by CPxplorer, including the instrument used for data acquisition, ∑PCAs-$C_{10-13}$, ∑PCAs-$C_{14-17}$, ∑PCAs-$C_{18-30}$ and the goodness fit of the nnls model. The results presented are the averages of three replicates, with the standard deviation shown as the ± error.

| Instrument | ∑PCAs-$C_{10-13}$ (µg/g) | ∑PCAs-$C_{14-17}$ (µg/g) | ∑PCAs-$C_{18-30}$ (µg/g) | nnls fit ($R^2$) |
|---|---|---|---|---|
| DI-APCI-Orbi | 6.6±0.2 | 13.5±0.6 | 12.5±0.7 | 0.94±0.00 |
| LC-APCI-Orbi | 11±1 | 24±2 | 25±1 | 0.93±0.01 |
| LC-ESI-qToF | 11±1 | 18±4 | 20±4 | 0.78±0.00 |
| GC-NCI-Orbi | 6.9±1.7 | 2.5±0.6 | - | 0.92±0.03 |
| GC-ECNI-qToF | 6.1±0.2 | 3.1±0.2 | - | 0.95±0.01 |

***Table S3:*** Quantification results for the matrices used to address human exposure (IOF, indoor dust, SWB, meat and vegetables) obtained by CPxplorer, including the ∑PCAs-$C_{10-13}$, ∑PCAs-$C_{14-17}$, ∑PCAs-$C_{18-30}$ and the goodness fit of the nnls model. Data was acquired using LC-ESI-qToF for all the matrices.

| Matrix | ∑PCAs-$C_{10-13}$ | ∑PCAs-$C_{14-17}$ | ∑PCAs-$C_{18-30}$ | nnls fit ($R^2$) |
|---|---|---|---|---|
| IOF (ng/$m^2$) | 147.5 | 268.2 | 175.2 | 0.50 |
| Dust (µg/g) | 6.9 | 22.3 | 6.3 | 0.90 |
| SWB (µg/g, w/w) | 1.1 | 5.6 | 1.0 | 0.40 |
| Meat (ng/g, w/w) | 53.1 | 25.1 | 5.1 | 0.60 |
| Vegetables (ng/g, w/w) | 19.7 | 9.5 | 1.8 | 0.63 |

***Table S4:*** Overview of polychlorinated alkanes (PCAs), transformation products derived from PCAs, polychlorinated olefins (PCOs) and bromochloro alkanes (BCAs). The table includes the compound family, corresponding general formulas, example molecular formulas, example adduct ions, and the calculated exact masses of the monoisotopic adduct ions that were obtained using EnviPat.[8]

| Family | Transformation product | General formula | Example formula | Example adduct ion | Example adduct ion m/z (calculated exact mass of the monoisotopic adduct ion of the transformation product using www.envipat.eawag.ch) |
|---|---|---|---|---|---|
| PCA | None | $C_xH_{2x+2-y}Cl_y$ | $C_{10}H_{16}Cl_6$ | $[M - H]^-$ | 344.93104 |
| Hydrolysis of PCA | -Cl+OH | $C_xH_{2x+2-y+1}Cl_{y-1}O$ | $C_{10}H_{17}Cl_5O$ | $[M - H]^-$ | 326.9649 |
| Double hydrolysis of PCA | -2Cl+2OH | $C_xH_{2x+2-y+2}Cl_{y-2}O_2$ | $C_{10}H_{18}Cl_4O_2$ | $[M - H]^-$ | 308.9988 |
| PCA- hydroxylation | -H+OH | $C_xH_{2x+2-y}Cl_yO$ | $C_{10}H_{16}Cl_6O$ | $[M - H]^-$ | 360.9295 |
| Double PCA- hydroxylation | -2H+2OH | $C_xH_{2x+2-y}Cl_yO_2$ | $C_{10}H_{16}Cl_6O_2$ | $[M - H]^-$ | 376.92087 |
| PCA-oxidation to ketone | -2H+O | $C_xH_{2x+2-y-2}Cl_yO$ | $C_{10}H_{14}Cl_6O$ | $[M - H]^-$ | 358.91030 |
| PCA- Sulfonation | $-H+SO_4H$ | $C_xH_{2x+2-y}Cl_yO_4S$ | $C_{10}H_{16}Cl_6O_4S$ | $[M - H]^-$ | 440.88277 |
| PCA- Glucuronidation | $-H+C_6H_{10}O_7$ | $C_{x+6}H_{2x+2-y+9}Cl_yO_7$ | $C_{16}H_{25}Cl_6O_7$ | $[M - H]^-$ | 537.96587 |
| PCO | None | $C_xH_{2x-y}Cl_y$ | $C_{10}H_{14}Cl_6$ | $[M - H]^-$ | 342.91539 |
| BCA | None | $C_xH_{2x+2-y-z}Cl_yBr_z$ | $C_{10}H_{15}Cl_6Br$ | $[M - Cl]^-$ | 388.880526 |

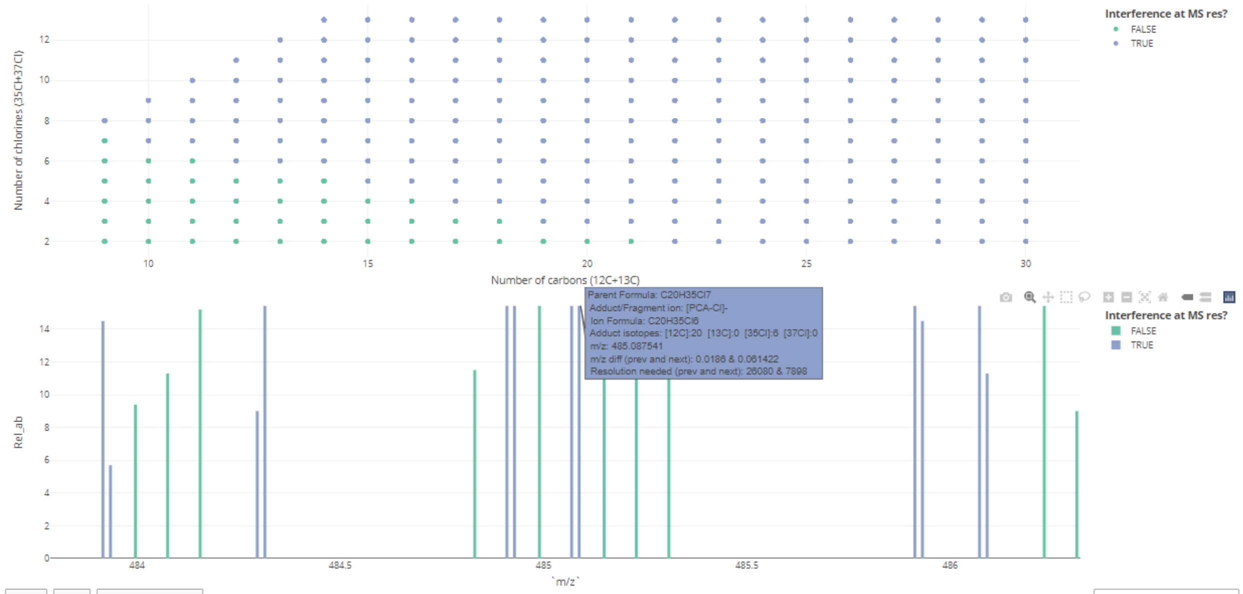


| Parent_Formula | Cl_perc | Charge | Adduct | Adduct_Formula | Isotopologue | Isotope_Formula | m/z | Rel_ab | 12C | 13C | 1H | 2H | 35Cl | 37Cl |
|---|---|---|---|---|---|---|---|---|---|---|---|---|---|---|
| C9H15Cl3 | 46.33 | -1 | [PCO-Cl]- | C9H15Cl2 | | [12C]9[13C]0[1H]15[2H]0[35Cl]2[37Cl]0 | 193.055629 | 100 | 9 | 0 | 15 | 0 | 2 | |
| C9H15Cl3 | 46.33 | -1 | [PCO-Cl]- +1 | C9H15Cl2 | +1 | [12C]8[13C]1[1H]15[2H]0[35Cl]2[37Cl]0 | 194.058984 | 9.7 | 8 | 1 | 15 | 0 | 2 | |
| C9H15Cl3 | 46.33 | -1 | [PCO-Cl]- +2 | C9H15Cl2 | +2 | [12C]9[13C]0[1H]15[2H]0[35Cl]1[37Cl]1 | 195.052679 | 64 | 9 | 0 | 15 | 0 | 1 | |
| C9H17Cl3 | 45.92 | -1 | [PCA-Cl]- | C9H17Cl2 | | [12C]9[13C]0[1H]17[2H]0[35Cl]2[37Cl]0 | 195.07128 | 100 | 9 | 0 | 17 | 0 | 2 | |
| C9H15Cl3 | 46.33 | -1 | [PCO-Cl]- +3 | C9H15Cl2 | +3 | [12C]8[13C]1[1H]15[2H]0[35Cl]1[37Cl]1 | 196.056034 | 6.2 | 8 | 1 | 15 | 0 | 1 | |
| C9H17Cl3 | 45.92 | -1 | [PCA-Cl]- +1 | C9H17Cl2 | +1 | [12C]8[13C]1[1H]17[2H]0[35Cl]2[37Cl]0 | 196.074634 | 9.7 | 8 | 1 | 17 | 0 | 2 | |
| C9H15Cl3 | 46.33 | -1 | [PCO-Cl]- +4 | C9H15Cl2 | +4 | [12C]9[13C]0[1H]15[2H]0[35Cl]0[37Cl]2 | 197.049729 | 10.2 | 9 | 0 | 15 | 0 | 0 | |
| C9H17Cl3 | 45.92 | -1 | [PCA-Cl]- +2 | C9H17Cl2 | +2 | [12C]9[13C]0[1H]17[2H]0[35Cl]1[37Cl]1 | 197.068329 | 64 | 9 | 0 | 17 | 0 | 1 | |
| C9H17Cl3 | 45.92 | -1 | [PCA-Cl]- +3 | C9H17Cl2 | +3 | [12C]8[13C]1[1H]17[2H]0[35Cl]1[37Cl]1 | 198.071684 | 6.2 | 8 | 1 | 17 | 0 | 1 | |
| C9H17Cl3 | 45.92 | -1 | [PCA-Cl]- +4 | C9H17Cl2 | +4 | [12C]9[13C]0[1H]17[2H]0[35Cl]0[37Cl]2 | 199.065379 | 10.2 | 9 | 0 | 17 | 0 | 0 | |
| C10H17Cl3 | 43.66 | -1 | [PCO-Cl]- | C10H17Cl2 | | [12C]10[13C]0[1H]17[2H]0[35Cl]2[37Cl]0 | 207.07128 | 100 | 10 | 0 | 17 | 0 | 2 | |
| C10H17Cl3 | 43.66 | -1 | [PCO-Cl]- +1 | C10H17Cl2 | +1 | [12C]9[13C]1[1H]17[2H]0[35Cl]2[37Cl]0 | 208.074634 | 10.8 | 9 | 1 | 17 | 0 | 2 | |

***Figure S2:*** Interfering ion results GUI display from CPions. Ions that do not exhibit any interference are depicted in green, while those causing interference are shown in blue. The figure includes overlapping based on molecular formula, overlapping based on m/z values, and a summary of the results presented in a table, from top to bottom.

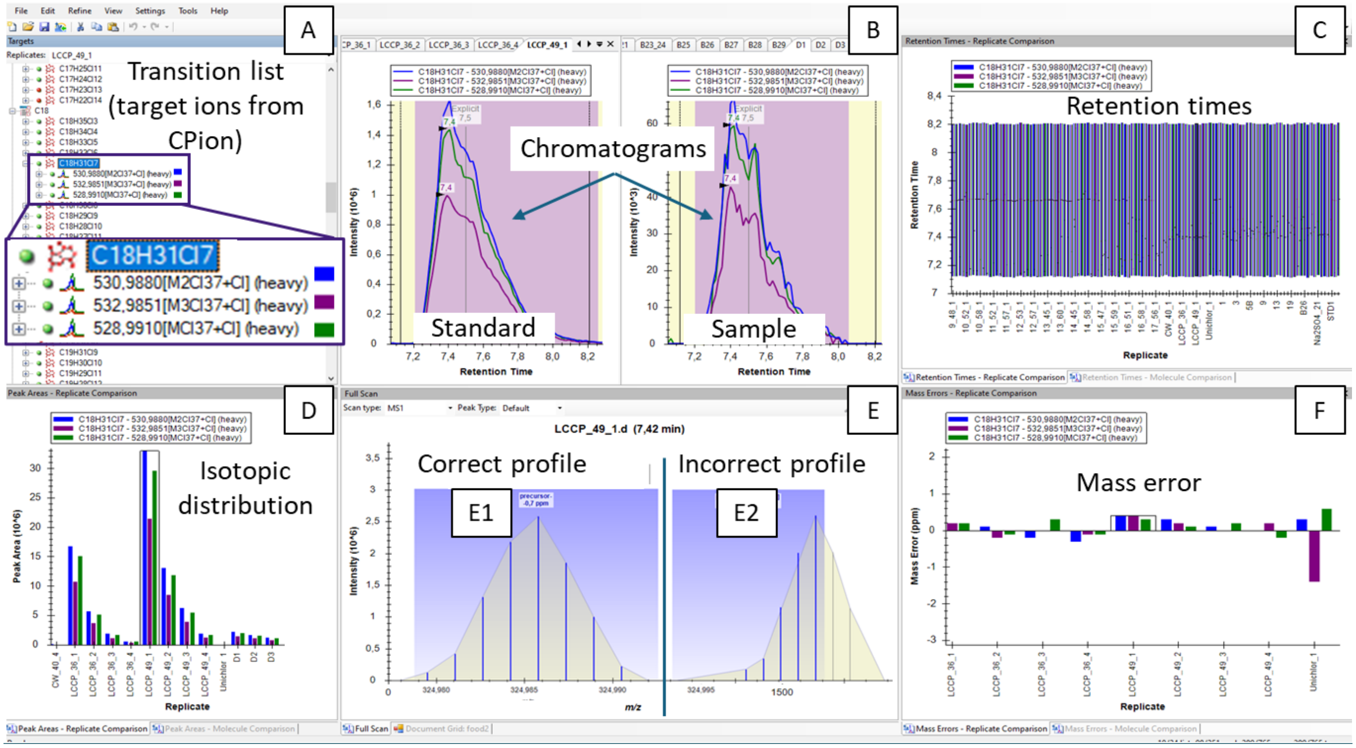


***Figure S3:*** Skyline GUI display for PCA data analysis. A-F depict schematically the data integration criteria, where (A) shows the list of target ions imported from the CPions-generated target list, the displayed example (targeted three ions for $C_{18}H_{31}Cl_7$) is zoomed in, (B) the extracted ion chromatograms (EIC) for these three ions of the same homologue group for ($C_{18}H_{31}Cl_7$) for the standard and the sample, (C) the retention time for three ions of the same homologue group ($C_{18}H_{31}Cl_7$), (D) the peak area for three ions of the same homologue group ($C_{18}H_{31}Cl_7$) which displays the isotopic distribution, (E) Full scan panel were the MS data can be displayed in profile or centroided mode, in blue the targeted ions, (E1) shows the profile mode MS data corresponding to the EIC of the standard (the standard and the sample are displayed individually), as an example (E2) illustrates some profile data that does not fulfill the identification criteria, (F) the mass error for three ions of the same homologue group ($C_{18}H_{31}Cl_7$).

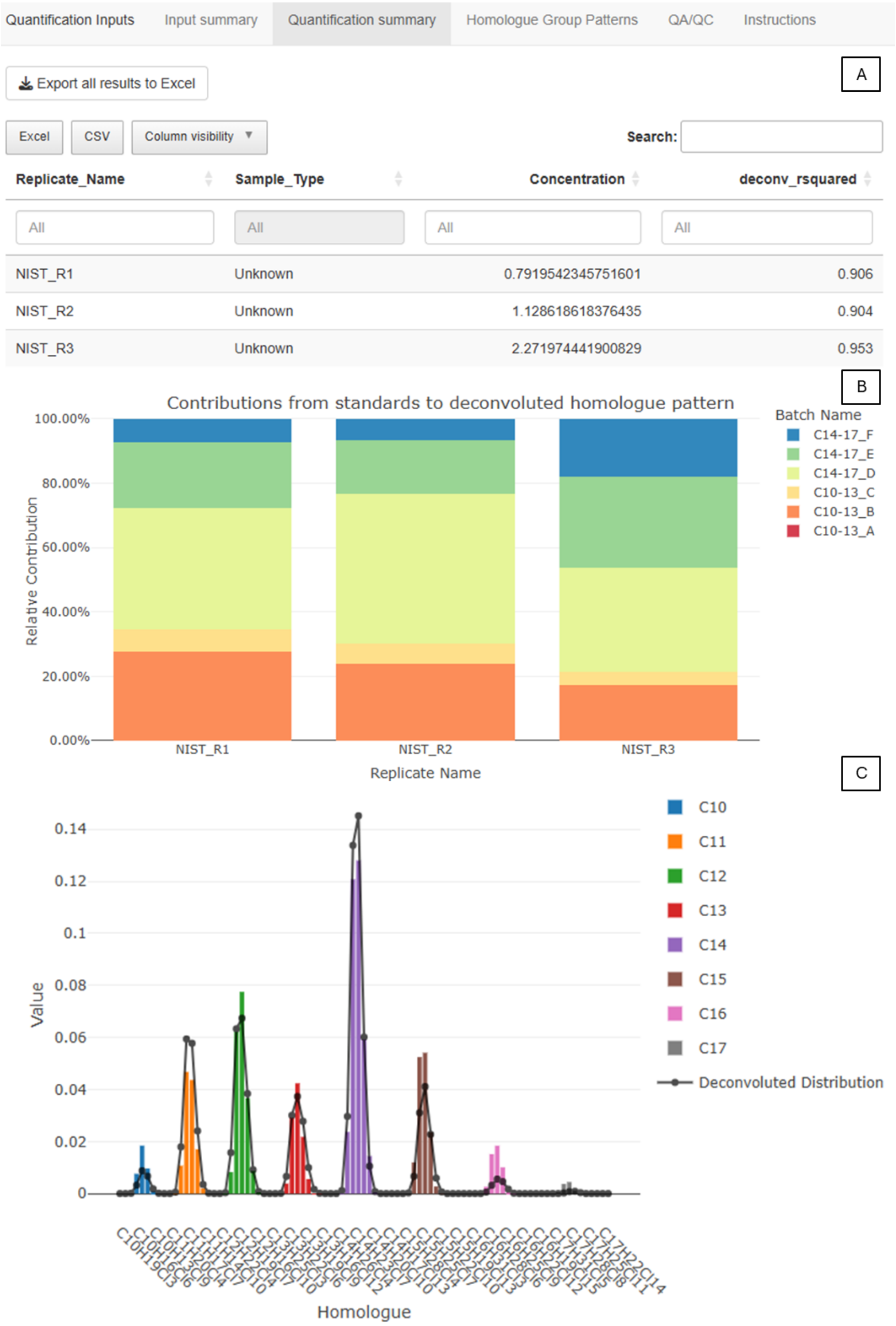


***Figure S4:*** CPquant GUI display for PCA quantification. (A) Overview of the quantification results for three samples, including “Replicate_Name” which shows the

name given to the data file in Skyline, “Sample_Type” which shows either if it is a sample (unknwon) or a blank, “Concentration” which shows the ∑PCAs quantified in the sample (the unit is equal to the one imputed by the user to build the calibration curves), and the “deconv_rsquared” which shows the goodness of fit between the measured and reconstituted patterns ($R^2$); (B) displays the different contributions of the standards to build the reconstituted patterns of the samples (the codes of the standards correspond to the ones imputed in Skyline); and (C) displays the PCA homologue pattern measured and reconstituted in one of the samples, where the bars show the measured one and the line depicts the nnls reconstituted one based on the deconvolution method [4,5].

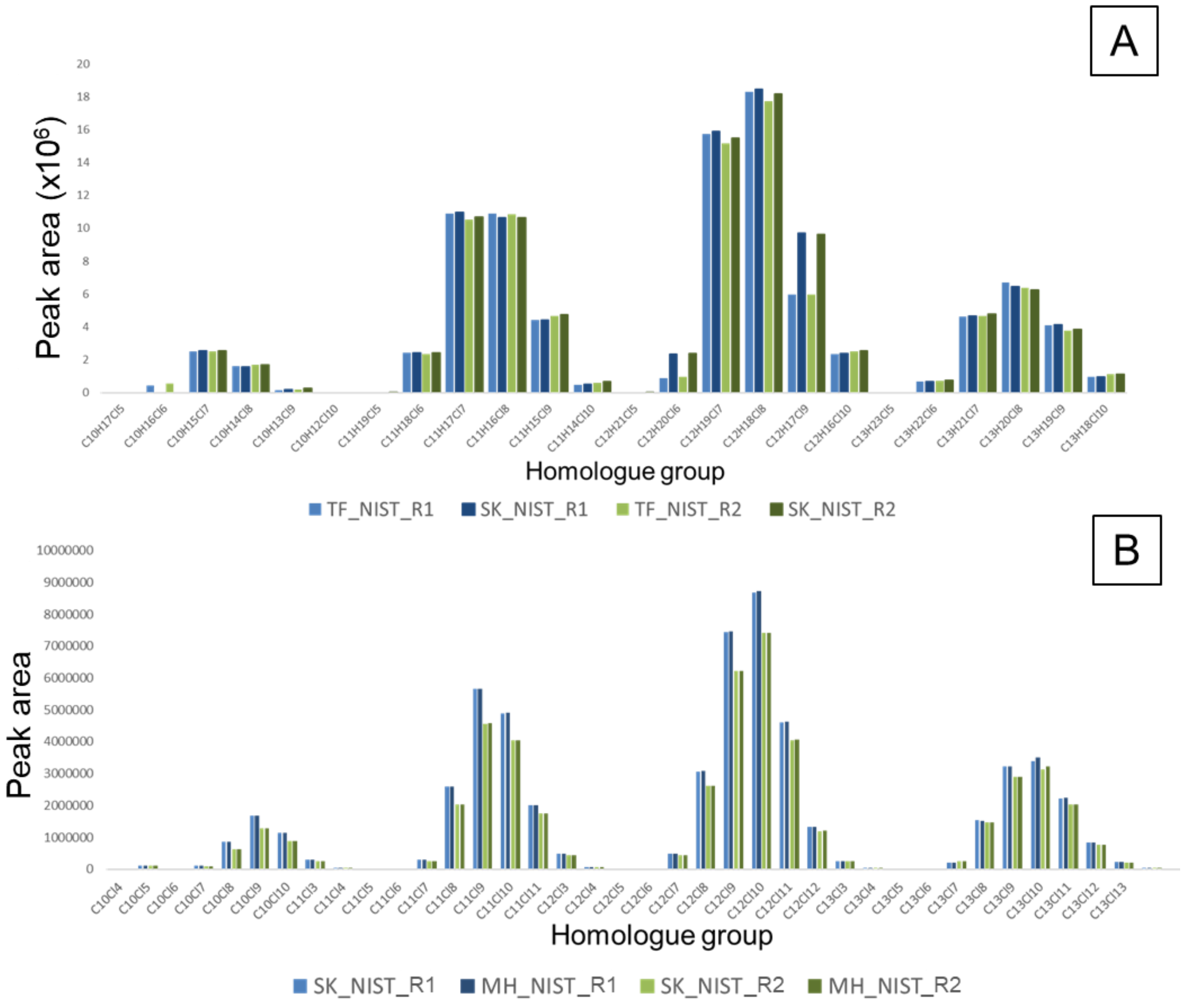


***Figure S5:*** Peak area of the base peak for PCA-$C_{10-13}$ homologue groups from two replicated of NIST-SRM-2585 indoor dust, integrated using (A) Trace Finder (TF), and Skyline (SK) for GC-NCI-Orbitrap data; (B) Skyline (SK) and Mass Hunter (MH) for LC-ESI-qToF data. Note: the suffix “_R1” refers to same replicate (depicted in blue in the online version), and “_R2” refers to the other replicate (depicted in green in the online version).

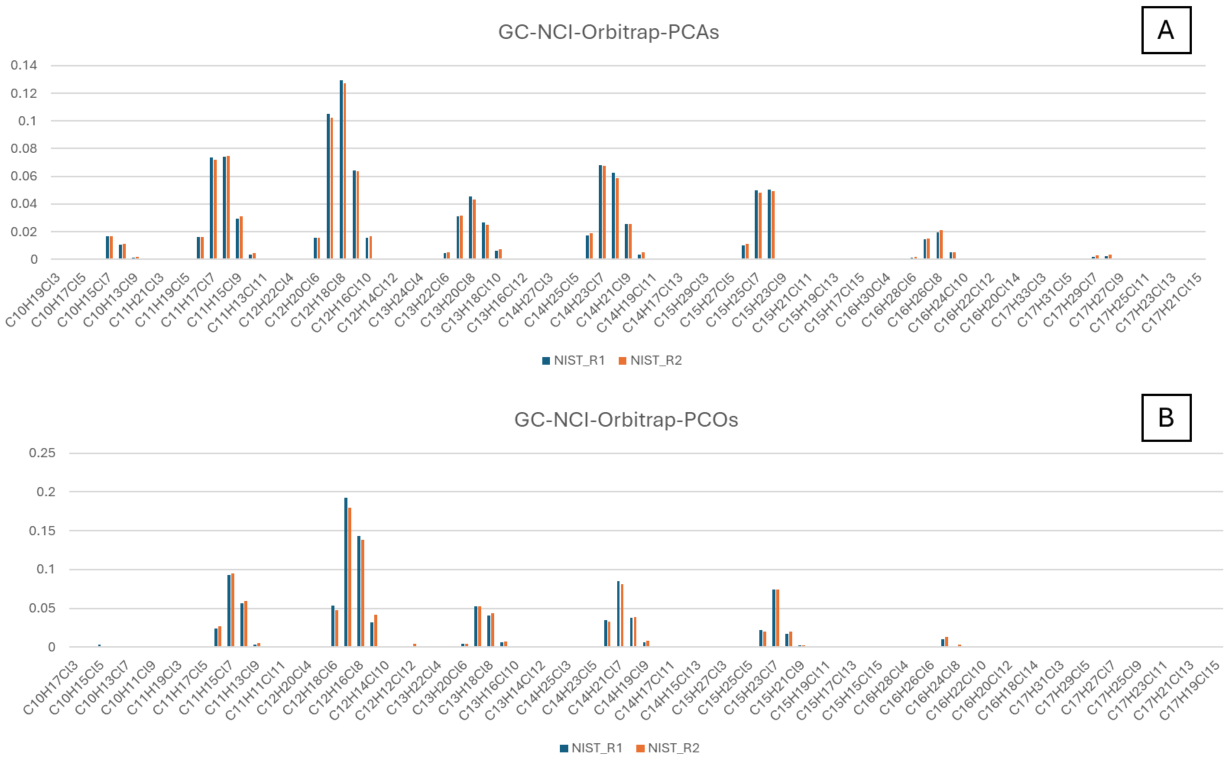


***Figure S6:*** Relative distribution of (A) PCAs-$C_{10-17}$ and (B) PCOs-$C_{10-17}$ measured in the NIST-SRM-2585 indoor dust by GC-NCI-Orbitrap. The relative distribution was calculated based on the instrumental response which was analyzed by CPxplorer using the modules CPions and Skyline. NIST_R1 and NIST_R2 are two replicates of NIST-SRM-2585.

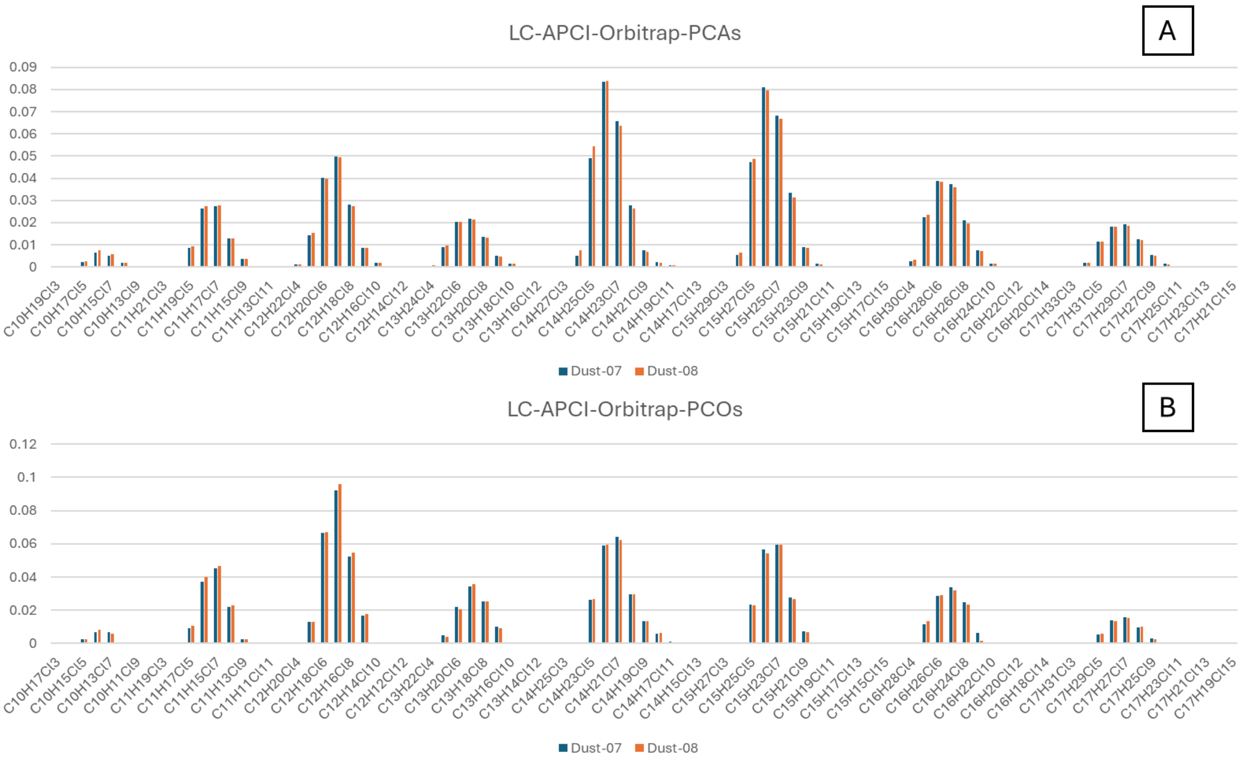


***Figure S7:*** *Relative* distribution of (A) PCAs-$C_{10-17}$ and (B) PCOs-$C_{10-17}$ measured in the NIST-SRM-2585 indoor dust by LC-APCI-Orbitrap. The relative distribution was calculated based on the instrumental response which was analyzed by CPxplorer using the modules CPions and Skyline. Dust 07 and 08 are two replicates of NIST-SRM-2585.

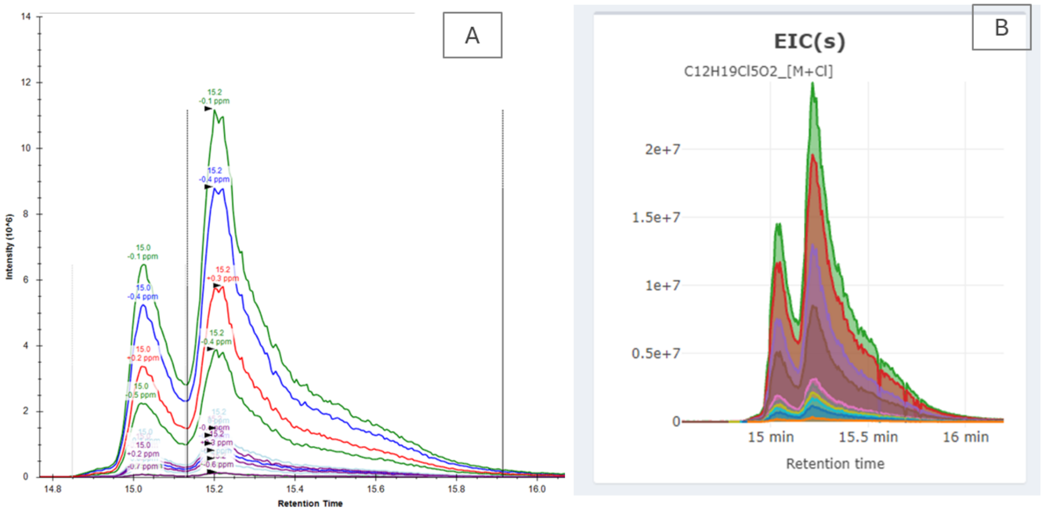


***Figure S8:*** Extracted ion chromatogram of $C_{12}H_{19}Cl_5O_2$ $[M + Cl]^-$ (A) by CPxplorer using the CPions and Skyline modules, and (B) by CP-Seeker. The data was acquired by UPLC-ESI-Orbitrap for rice plant roots (*Oryza sativa* Japonica cv. Nipponbare) previously exposed to1,2,5,6,9,10-$C_{10}H_{16}Cl_6$ by Chen et al. (2022).[6]

## REFERENCES


1. Spaan, K. M., Yuan, B., Plassmann, M. M., Benskin, J. P. & de Wit, C. A. Characterizing the Organohalogen Iceberg: Extractable, Multihalogen Mass Balance Determination in Municipal Wastewater Treatment Plant Sludge. *Environ. Sci. Technol.* **57**, 9309–9320 (2023).

2. Ricci, M. *et al.* Stepping-up accurate quantification of chlorinated paraffins: Successful certification of the first matrix reference material. *Anal. Chim. Acta* **1315**, (2024).

3. Livsmedelsverket. *Swedish Market Basket Survey 2015-per Capita-Based Analysis of Nutrients and Toxic Compounds in Market Baskets and Assessment of Benefit or Risk*. (2017).

4. Bogdal, C., Alsberg, T., Diefenbacher, P. S., Macleod, M. & Berger, U. Fast quantification of chlorinated paraffins in environmental samples by direct injection high-resolution mass spectrometry with pattern deconvolution. *Anal. Chem.* **87**, 2852–2860 (2015).

5. Perkons, I., Pasecnaja, E. & Zacs, D. The impact of baking on chlorinated paraffins: Characterization of C10–C17 chlorinated paraffins in oven-baked pastry products and unprocessed pastry dough by HPLC–ESI–Q–TOF–MS. *Food Chem.* **298**, (2019).

6. Chen, W. *et al.* Biotic and Abiotic Transformation Pathways of a Short-Chain Chlorinated Paraffin Congener, 1,2,5,6,9,10-C10H16Cl6, in a Rice Seedling Hydroponic Exposure System. *Environ. Sci. Technol.* **56**, 9486–9496 (2022).

7. McGrath, T. J. *et al.* Detection of Bromochloro Alkanes in Indoor Dust Using a Novel CP-Seeker Data Integration Tool. *Anal. Chem.* (2023) doi:10.1021/acs.analchem.3c05800.

8. Loos, M., Gerber, C., Corona, F., Hollender, J. & Singer, H. Accelerated isotope fine structure calculation using pruned transition trees. *Anal. Chem.* **87**, 5738–5744 (2015).
